# Towards accurate and efficient diagnoses in nephropathology: An AI-based approach for assessing kidney transplant rejection

**DOI:** 10.1016/j.csbj.2024.08.011

**Published:** 2024-08-16

**Authors:** Alexey Fayzullin, Elena Ivanova, Victor Grinin, Dmitry Ermilov, Svetlana Solovyeva, Maxim Balyasin, Alesia Bakulina, Pavel Nikitin, Yana Valieva, Alina Kalinichenko, Alexander Arutyunyan, Aleksey Lychagin, Peter Timashev

**Affiliations:** a Institute for Regenerative Medicine, Sechenov First Moscow State Medical University (Sechenov University), 8–2 Trubetskaya st., Moscow 119991, Russia; b World-Class Research Center “Digital Biodesign and Personalized Healthcare, Sechenov First Moscow State Medical University (Sechenov University), 8–2 Trubetskaya st., Moscow 119991, Russia; c B.V.Petrovsky Russian Research Center of Surgery, 2 Abrikosovskiy lane, Moscow 119991, Russia; d PJSC VimpelCom, 10 8th March Street, Moscow 127083, Russia; e Scientific and Educational Resource Center, Peoples’ Friendship University of Russia, 6 Miklukho-Maklaya st., Moscow 117198, Russia; f Department of Trauma, Orthopedics and Disaster Surgery, Sechenov First Moscow State Medical University (Sechenov University), 8–2 Trubetskaya st., Moscow 119991, Russia

**Keywords:** Artificial intelligence, Transplant rejection, Digital pathology, Whole slide images, Computational pathology

## Abstract

The Banff classification is useful for diagnosing renal transplant rejection. However, it has limitations due to subjectivity and varying concordance in physicians' assessments. Artificial intelligence (AI) can help standardize research, increase objectivity and accurately quantify morphological characteristics, improving reproducibility in clinical practice. This study aims to develop an AI-based solutions for diagnosing acute kidney transplant rejection by introducing automated evaluation of prognostic morphological patterns. The proposed approach aims to help accurately distinguish borderline changes from rejection. We trained a deep-learning model utilizing a fine-tuned Mask R-CNN architecture which achieved a mean Average Precision value of 0.74 for the segmentation of renal tissue structures. A strong positive nonlinear correlation was found between the measured infiltration areas and fibrosis, indicating the model's potential for assessing these parameters in kidney biopsies. The ROC analysis showed a high predictive ability for distinguishing between ci and i scores based on infiltration area and fibrosis area measurements. The AI model demonstrated high precision in predicting clinical scores which makes it a promising AI assisting tool for pathologists. The application of AI in nephropathology has a potential for advancements, including automated morphometric evaluation, 3D histological models and faster processing to enhance diagnostic accuracy and efficiency.

## Graphical Abstract

**Figure:**
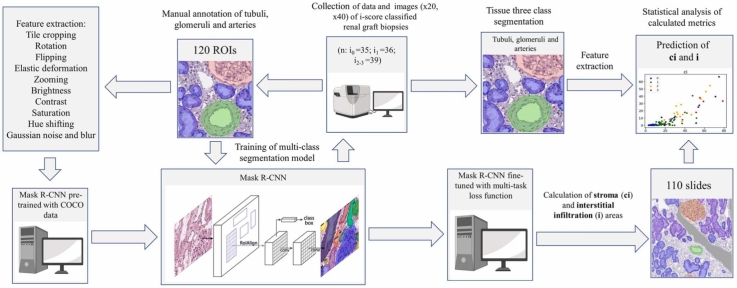
Study design: The model was trained on 120 annotated regions of interest (ROIs) of transplanted kidneys, containing manually annotated renal tissue structures such as glomeruli, tubules and arteries. We utilized the Mask R-CNN architecture known for its precise object selection in images. The Mask R-CNN model was pretrained on COCO and further trained through an original multi-task loss function, incorporating manually segmented ROIs. Following training, model generated multi-class segmentation masks for 110 histological slide images. These masks were used to calculate relative areas for the stromal component of the kidney and interstitial infiltration. Extracted metrics were then analyzed for correlations with fibrosis (ci) and interstitial infiltration (i) expert scores based on the Banff classification.

## 1 Introduction

Kidney transplantation is considered the most optimal treatment strategy for chronic kidney failure, significantly prolonging life and improving its quality for patients [1]. The first successful kidney transplantation surgery was performed in 1954 by American surgeon Joseph Murray [2]. The number of kidney transplant surgeries reached a record level of 22,393 in the US in 2018, increasing by 6.5 % compared to 2017 [3].

The lifespan of the transplant depends on many factors, which can be reliably assessed through morphological research. The loss of the transplant function can be caused by both rejection and recurrence of the underlying disease. Rejection remains the predominant cause of transplant failure despite the progress in immunosuppressive therapy. Rejection is considered an immunological reaction of the recipient to donor antigens, which leads to the destruction of transplanted tissue or organ. It is divided into two major subgroups based on the mechanism of pathogenesis: T-cell-mediated rejection and antibody-mediated rejection. In some cases, rejection has a mixed nature. Rejection can also be acute or chronic, depending on the disease progression [4]. Acute rejection develops in 7.0 % of adult recipients within the first year after transplantation with the incidence of 9.1 % in recipients aged 18–34 % and 6.1 % in recipients over 65 [5]. Histological examination of biopsies is the primary laboratory approach to diagnose rejection in a hospital.

Several morphological classifications have been developed, primarily related to acute cellular rejection. The Banff classification, in particular, is the most widely used by pathologists. This classification was first proposed by Kim Solez and Lorraine C. Racusen in 1991 and has since been revised several times [6]. The 2019 edition of the Banff classification indicates the following features as essential for the prognosis: fibrosis [ci], interstitial inflammation [i], tubulitis [t], arteritis [v] forcellular rejection; glomerulitis [g], peritubular capillaritis [ptc] and C4d deposition [C4d] for humoral rejection [6]. Each feature is given a score (ranging from 0 to 3 points), which is used by the pathologist to diagnose the graft status.

Despite its comprehensiveness, the Banff classification has some limitations. The most important limitation is the interobserver variability: the assessment of rejection severity in a biopsy can vary from physician to physician depending on their qualifications and of morphological findings. The Banff classification has a fairly large number of criteria, the assessment of which is based on a semi-quantitative method of scores.This method can be subjective, as pathologists must mentally calculate relative areas and relative numbers of findings, leading to potential differences in evaluations. Borderline changes are a particularly challenging diagnostic feature because patients with moderate pathological changes in the graft tissue receive different treatment than those with rejection [7]. The diagnostic criteria for this group revised twice in the Banff classification [7].

These issues can be addressed through the use of artificial intelligence (AI). AI can help standardize research, eliminate differences between observers and increase objectivity by accurately quantifying morphological characteristics. The latter argument is important for clearly distinguishing transplant rejection from borderline changes. Additionally, AI can help develop clear criteria based on program calculations algorithmic calculations, improving the reproducibility of the Banff classification in clinical practice.

Nephropathology is at the forefront of digital pathology revolution. AI has been intensively used for segmentation of structures in renal biopsies and their quantification. Convolutional neural networks (CNN) were applied to differentiate between normal and pathological tissues in whole slide images (WSI) [8], [9]. Subsequently, AI technologies became more sophisticated and were applied to assess the severity of pathological changes [8], [10]. Researchers are using AI to evaluate the extent of fibrosis in the interstitium and atrophy of renal tubules in chronic kidney disease. Automated evaluation of tissue vascularization and immune cell infiltration was used to assess inflammation in parenchymal and stromal parts of kidney [8], [10], [11], [12].

Our project is aimed at developing an AI-based and practice-oriented solution for the diagnosis of acute rejection in kidney transplant recipients. In the present study, we explored whether AI can accurately calculate the most important prognostic morphological patterns, fibrosis and interstitial infiltration, to predict expert scores and differentiate borderline changes from rejection. To achieve precise and reproducible results, we developed and validated a neural network for automated pre-classification of kidney transplant biopsies on WSI stained with hematoxylin and eosin.

## 2 Materials and methods

### 2.1 Dataset preparation

Histological slides of renal graft biopsies (n = 120) were found in the biobank of the Pathology Department of Petrovsky National Research Center of Surgery (Moscow, Russia). Our cohort consisted of 120 patients, including 74 men (61.66 %) and 46 women (38.34 %). The mean age of the patients was 39 ± 10.28 years, with ages ranging from 18 to 74 years. All patients were monitored in the Department of Kidney Transplantation at B.V. Petrovsky Russian Research Center of Surgery, with diagnoses related to complications of kidney transplant (ICD-10: T86.1). Inclusion criteria: (1) patient age over 18 years; (2) confirmed diagnosis of T-cell mediated rejection in anamnesis. Exclusion criteria: (1) presence of nephrosclerosis in the specimens; (2) clinical and/or morphological signs of antibody mediated rejection; (3) presence of artifacts in the image. We included nephrosclerosis in our exclusion criteria because it involves the replacement of kidney parenchyma with connective tissue, making a reliable detection of rejection impossible. Mechanisms of nephrosclerosis are significantly different from transplant rejection and include recurrence of the disease, hypertensive angiosclerosis and inflammatory conditions like pyelonephritis or viral infections. All slides were prepared from percutaneous biopsies that were fixed in formalin, underwent standard histological processing, were embedded into paraffin blocks, sectioned and stained with hematoxylin and eosin. The search for the renal graft slides was conducted through laboratory information system which allowed to get a balanced selection of cases based on an interstitial infiltration score (35 slides of i = 0; 36 slides of i = 1; 39 slides of i = 2–3). Slides were prepared between 2004 and 2022 according to the data in the information system (Table 1). Cases of nephrosclerosis were excluded from the study. We scanned the samples with Leica Aperio AT2 at x20 (n = 29) and x40 (n = 81) magnifications. The decision to scan at different magnifications was made to ensure that the model would be accurate in hospitals prioritizing velocity of the routine analysis since this was the case in the Center where we obtained the images. All WSI were anonymized and did not contain labels referring to clinical cases. We randomly divided the patient identifiers into two mutually exclusive sets, for training and validation, in order to prevent data leakage. This method guaranteed that the WSIs in the training set were entirely independent of those in the validation set, thereby providing a robust assessment of the model's generalizability to unseen data. Two pathologists with significant experience of studying renal grafts (8 and 30 years) reevaluated the slides using 2019 revision of the Banff classification (still relevant after 2022 Banff classification update). The reevaluation was done blindly using WSIs of.svs format in CaseViewer (3DHistech, Hungary).

**Table 1:** Expert scores for fibrosis (ci) and interstitial infiltration (i) findings in whole slide images of the validation dataset reevaluated according to 2019 revision of the Banff classification.

	**Number of slides**	
	**fibrosis ci**	**interstitial infiltration i**
**Magnification x20 (pixel size=0.5056 µm, tile size=250 ×250)**		
score = 0	18	13
score = 1	8	12
score = 2	3	3
score = 3	0	1
Total	29	29
**Magnification x40 (pixel size=0.2519 µm, tile size=500 ×500)**		
score = 0	30	22
score = 1	33	24
score = 2	14	32
score = 3	4	3
Total	81	81
Grand Total	110	110

Two pathologists annotated all tissue structures in 10 WSI with 2–3 sections of transplanted kidneys in QuPath v.0.4.3. 120 most different images (512 ×512 pixels, or 256 ×256 micrometer) were cropped and used to train the model and test its performance.

In 110 WSIs of the validation dataset, regions of interest were selected for automatic segmentation in QuPath v.0.4.3. The regions included renal cortex of one or multiple tissue sections and excluded the capsule and renal medulla.

The Dice score is used to evaluate the similarity between a predicted segmentation mask and the ground truth segmentation mask. The Dice score ranges from 0, indicating no overlap, to 1, indicating perfect overlap.

$$\mathrm{Dice Score}=\;\frac{2*\mathrm{TP}}{2*\mathrm{TP}+\mathrm{FN}+\mathrm{FP}}$$

where TP is the number of true positives, TN is the number of true negatives, FP is the number of false positives, and FN is the number of false negatives. Expected ranges of Dice score for good, acceptable and bad score could be > 0.8, 0.6–0.8 and < 0.6 respectively. For statistical analysis, we calculated t-statistic.

For the inter-observer agreement study, we used 20 images that were not included in the train set. Pathologists made additional annotations in the regions of interests in WSIs that were totally annotated by their colleagues.

### 2.2 Neural network architecture and training process

The deep learning model was trained on a dataset consisting of 52 training images, with data augmentation techniques applied to expand the effective dataset size. To mitigate overfitting and ensure robust model performance, we employed 20-fold cross-validation. The final model was evaluated on a separate test set of 68 images.

The following predefined classes were used for segmentation: "glomeruli," "tubuli", "arteries", "stroma", and "infiltration" (Figure A1).

For the segmentation of "glomeruli," "tubuli", and "arteries", we selected the Mask R-CNN architecture due to its proven effectiveness in instance segmentation tasks, particularly in medical image analysis. We utilized the Detectron2 framework for model implementation and training [13].

The Mask R-CNN model was initially pretrained on the COCO (Common Objects in Context) dataset. This pretraining step is crucial as it allows the model to learn general features from a large, diverse dataset, which can then be fine-tuned for our specific histopathological segmentation task. The COCO dataset was chosen because it provides a rich set of annotated images for object detection and segmentation, which aligns well with our task of identifying and segmenting specific structures in histopathological images.

We fine-tuned the pretrained model using the original multi-task loss function:

L = L_cls + L_box + L_mask

where L_cls is the classification loss, L_box is the bounding box regression loss, and L_mask is the mask prediction loss. This multi-task loss function enables the model to simultaneously learn to classify objects, refine their bounding boxes, and generate precise segmentation masks.

For optimization, we employed the Stochastic Gradient Descent (SGD) algorithm with a learning rate of 0.003. SGD was chosen for its effectiveness in training deep neural networks and its ability to escape local minima through stochastic updates. The specific learning rate of 0.003 was determined through empirical tuning, balancing the trade-off between convergence speed and stability. This relatively low learning rate helps prevent overshooting during optimization and allows for more fine-grained updates to the model parameters.

On each of the 20 cross-validation folds, the Mask R-CNN model was trained for 300 epochs with a batch size of 16. The batch size was chosen to balance computational efficiency and model performance, considering the available GPU memory and the complexity of the segmentation task.

To enhance the model's generalization capabilities and account for variations in tissue morphology and staining, we employed extensive data augmentation techniques, which included geometric transformations (rotation, flipping, elastic deformation, zooming), color and intensity adjustments (brightness, contrast, saturation, hue shifting) and noise and blur (Gaussian noise and Gaussian blur).

These augmentation techniques help the model learn invariance to various image transformations and artifacts that may be present in histopathological images, thereby improving its robustness and performance on unseen data.

### 2.3 Segmentation performance

Mean Average Precision (mAP) is commonly used to analyze the performance of segmentation systems.The mAP is calculated by finding Average Precision (AP) for each segmentation class and then average over a number of classes.

$$\mathrm{mAP}=\frac{1}{N}\sum_{i=1}^{N}{\mathrm{AP}}_{i},$$

where parameter N is a number of classes.

To calculate AP, we plotted the precision-recall curve and calculated the area under it.

### 2.4 Slides processing

Primary biopsy tissue segmentation was done by applying light intensity thresholding on grayscale images. We used a sliding window to analyze the entire slide image because the neural network accepts a small tiles with a size of 50 micrometers (99 pixels for WSI with x20 magnification) as input. It should be noted that we discarded small instances on the border of the sliding window, because their classes were determined unstable. When the scanning was completed, we merged instances of the same class with a strong intersection. The output result of the model was a segmentation mask for three classes (“glomeruli,” “tubuli”, “arteries").

All tissues that have not been assigned to these classes were defined as stroma. In normal renal tissue samples, the stroma occupied a small space adjacent to each tissue structure, measuring approximately 15–20 micrometers (equivalent to 30 ×30–40 ×40 pixels in whole-slide images at 20x magnification). We visualized these areas as light blue in the segmentation mask. These areas were excluded when calculating metrics for fibrosis and interstitial infiltration. In cases with edema, the space around tissue structures mitigated the increase in the calculated relative area of stroma. Conversely, in areas of fibrosis, connective tissue replaced renal structures, reducing the presumably normal area around the structures and resulting in a high relative area of fibrosis. The search for nuclei and lymphocytes was carried out in the stroma space. Thresholding and watershed algorithm were used to identify lymphocytes. These round cells were selected by calculating ellipticity (an ellipse was calculated for each object). Further, the found objects were classified as nuclei or lymphocytes depending on the size and color. The ratio of the semi-axes for the lymphocytes was less than 2. The color parameter was measured in gray images, on a scale of 0–255. A threshold of 95 was applied for average lymphocyte brightness. Further, areas containing a large number of nuclei and lymphocytes were considered areas of infiltration. Infiltration was considered to be a 50 × 50 micrometer area (99 ×99 pixels for WSI with x20 magnification) containing 2 lymphocytes and 20 nuclei and more.

### 2.5 Morphological analysis

Slide image in the validation dataset (n = 110) with overlaying segmentation masks were studied by the pathologists. Segmentation of stromal components was analyzed for accurate predictions and mistakes of the model. The cases with the discrepancy between expert scores and automatic calculations were studied and described in the results section. For assessing generalizability of our model, we used 50 WSIs from CPTAC-CCRCC dataset as an independent external cohort [14]. In each slide, we selected one square ROI of non-tumorous renal tissue with a perimeter of 1024 micrometer for the annotation (Supplementary Material – verification.zip). The pathologists manually annotated “tubuli”, “glomeruli” and “arteries” as described earlier. We compared the results of segmentation with ground truth and evaluated the predictive accuracy of the algorithm by calculating the Dice score.

### 2.6 Statistical analysis

The statistical analysis of the experimental quantitative data was performed using GraphPad Prism version 8.00 for Windows (GraphPad Software, Inc., San Diego, CA, USA) and R v4.1.2, with the betareg v3.1–4, car v3.0–13 and emmeans v1.7.3 libraries. To evaluate the differences between groups (scores), a beta regression models were used followed by one-way ANOVA and post-hoc Tukey analysis. Model assumptions were checked using the performance v0.9.0 library. ROC analysis was performed to compare the features and Youden's J index was calculated. Two-dimensional visualization of the calculated values was done using Python v.3.10 and NumPy v.1.21.1 and pandas v.1.4.1 libraries. The statistical analysis results were presented as violin plots. Statistical significance was set at p < 0.05.

## 3 Results

### 3.1 Multiclass segmentation performance

We annotated three classes of tissue structures (“tubuli”, “glomeruli” and “arteries”). The remaining tissue formed the 4th ”stroma” class. Dice coefficient for annotations of two pathologists without annotation class was 0.9065 (Q2, Min-Max: 0.8291 – 0.9297).

**Fig. 1:**
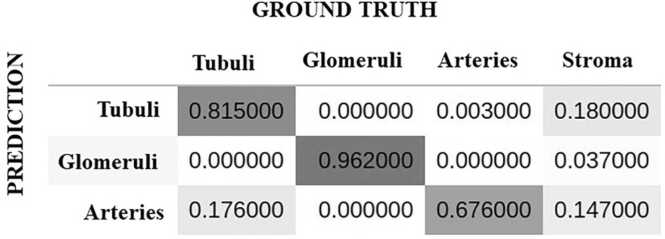
Confusion matrix for segmentation classes of the model.

We used a confusion matrix to visualize the performance of the model. Each column of the matrix represents the instances in an actual (ground truth) class while each raw represents the instances in a predicted class (Fig. 1). The volumes of the classes were approximately related as 40:3:2 (“tubuli”, “glomeruli” and “arteries”). We observed a small type 1 error 0.18 in the class “tubuli” in favor of “stroma” and type 1 errors 0.176 and 0.147 in the class “arteries” in favor of “tubuli” “and “stroma”. The best segmented class was “glomeruli” (0.962 accuracy). The performance of our model allowed to acquire high-quality predictions. However, the class predictions can be improved in further studies.

Our trained neural network showed value of mean Average Precision m〖AP〗([0.5:0.95])= 0.74 on the three classes task (“tubuli”, “glomeruli” and “arteries”). Dice coefficient was measured for the classes that were segmented by the AI model: tubuli - 0.8310, glomeruli - 0.9242, arteries - 0.7087, stroma - 0.7919.

### 3.2 Fibrosis and interstitial infiltration in percutaneous biopsy samples

**Fig. 2:**
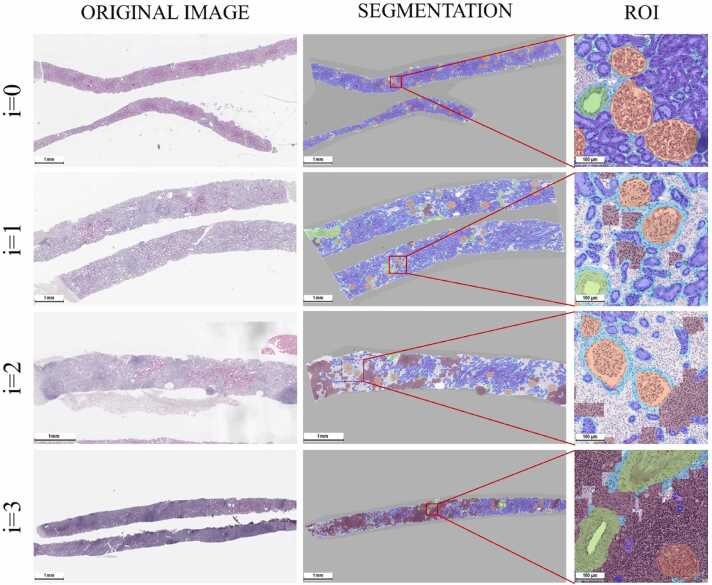
Segmentation of kidney tissue structures in whole slide images and selected regions of interest (ROIs). Interstitial infiltration (i) was visualized brown and represented a part of the stromal space that had a high density of lymphocytes. The area of interstitial infiltration occupied a larger part of the biopsy in cases with higher expert (i) scores. Glomeruli were visualized as orange, tubules – blue, arteries – green, stroma – grey, proper stromal space around tissue structures – light blue.

Semi-transparent segmentation masks overlayed original H&E images and allowed to assess precision of tissue class predictions from morphological perspective (Fig. 2, Supplementary material Video S1). There were no significant differences in the quality of segmentation masks between the slide images made on different magnifications.

Supplementary material related to this article can be found online at doi:10.1016/j.csbj.2024.08.011.

The following is the Supplementary material related to this article Video 1.

Video 1

Glomeruli (segmented as orange) were successfully segmented in their true borders which included both glomerular tuft and Bowman capsule and presented oval-shaped structures with an approximate diameter of 100–150 µm. It is interesting that the AI model segmented even some glomeruli that could be missed by pathologists due to their minor size.

Renal tubules (segmented as blue) presented a densely packed collection of polygonal structures. They merged in larger segmentation mask elements in some cases but more often had an approximate size of 30–50 µm. The tubules were successfully detected when they were sparsely located in the areas of fibrosis and interstitial infiltration.

Arteries (segmented as green) were detected in their outer borders and included the lumen. The program detected both small (around 100 µm in size) and significantly larger arteries some of which were only partially present in the biopsy.

Stroma (segmented as grey) was the space of the tissue that was located between the structures mentioned above. It is important to note that there is a normal volume of stromal tissue consisting of thin bundles of collagen fibers and small capillaries that form the space around parenchymal structures. This space was not evaluated in the assessment of fibrosis and was segmented as light blue.

Interstitial infiltration (segmented as brown) was a part of the stroma that satisfied criteria for both morphology of lymphocyte cells and their density in the region. These regions were close to absent in the i = 0 group and gradually increased in relative area in more clinically adverse groups.

### 3.3 CNN versus Banff classification system

**Fig. 3:**
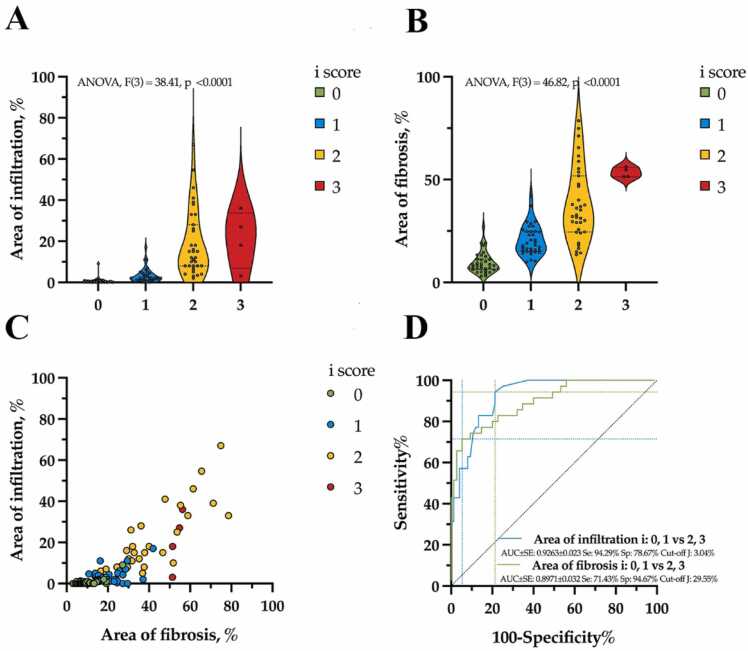
Statistical analysis of calculated relative areas of fibrosis and interstitial infiltration for interstitial infiltration (i) expert scores. (A, B) Comparative analysis of calculated fibrosis and interstitial infiltration measurements, beta regression model, analysis of variance (ANOVA), violin plot with dot-line medians and 25, 75 % percentile. (C) Distribution of the calculated parameters by scores. (D) Receiver operating characteristic (ROC) analysis of calculated parameters and cut-off estimation for scores.

**Fig. 4:**
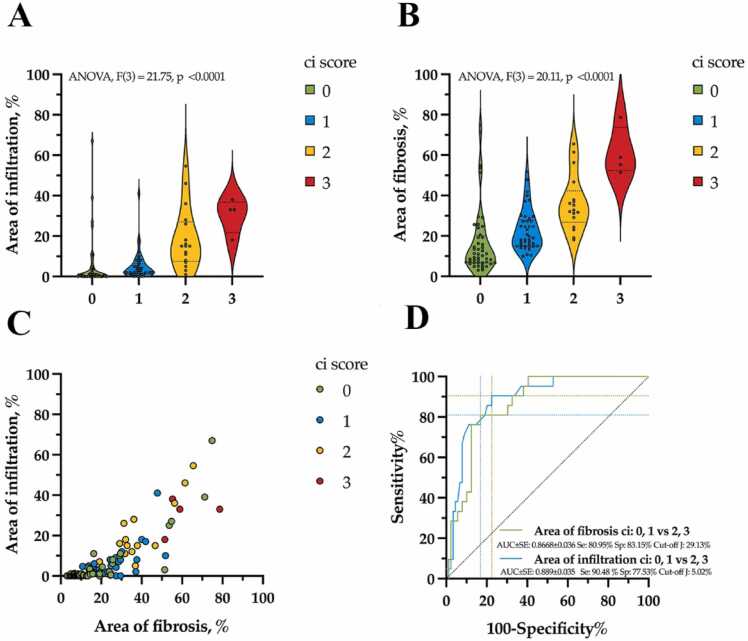
Statistical analysis of calculated relative areas of fibrosis and interstitial infiltration for fibrosis (ci) expert scores. (A, B) Comparative analysis of calculated fibrosis and interstitial infiltration measurements, beta regression model, analysis of variance (ANOVA), violin plot with dot-line medians and 25, 75 % percentile. (C) Distribution of the calculated parameters by scores. (D) Receiver operating characteristic (ROC) analysis of calculated parameters and cut-off estimation for scores.

A strong positive nonlinear correlation was found between the measured infiltration areas and fibrosis (Spearman r = 0.8212, p < 0.0001, Figs. 3C,4C).

There was a significant relationship between ci score and infiltration area (ANOVA, df=3, F=21.75, p < 0.0001, Table 2, Table 3, Fig. 3A) as well as between ci score and fibrosis area (ANOVA, df=3, F=20.11, p < 0.0001, Table 2, Table 3, Fig. 3B).

**Table 2:** Descriptive statistics on the measured areas of interstitial infiltration and fibrosis, according to the scores of the Banff classification system. Data in table includes median values with interquartile ranges (IQR) for infiltration and fibrosis across different Banff scores (0–3). Statistical significance (p values) derived from ANOVA tests are provided for both infiltration and fibrosis areas.

Score Measured area	Score 0	Score 1	Score 2	Score 3	p value (ANOVA)
**ci**	**n = 48**	**n = 41**	**n = 17**	**n = 4**	**Total 110**
infiltration	3.82 (2.43–5.21)	6.46 (4.27–8.64)	18.83 (12.61–25.05)	32.64 (16.43–48.85)	**<0.0001**
fibrosis	16.54 (13.51–19.57)	24.19 (20.32–28.06)	36.86 (29.97–43.75)	60.67 (46.27–75.08)	**<0.0001**
**i**	**n = 35**	**n = 36**	**n = 35**	**n = 4**	**Total 110**
infiltration	2.09 (1.24–2.95)	4.44 (2.87–6.01)	17.58 (13.91–21.25)	19.57 (8.48–30.67)	**<0.0001**
fibrosis	10.98 (8.61–13.35)	21.55 (18.33–24.78)	37.52 (33.6–41.45)	53.26 (41.27–65.26)	**<0.0001**

Beta regression model with µ (95 % CI) values; bold p values are significant at p < 0.05

**Table 3:** Comparison of mean differences in infiltration and fibrosis areas across scores of the Banff classification system (95 % CI). The table shows mean differences with 95 % confidence intervals (CI) and corresponding p-values (significant at p < 0.05 in bold) for pairwise comparisons between scores 1, 2, and 3 relative to score 0 within the interstitial infiltration and fibrosis for ci and i groups. Statistical analysis was conducted using post-hoc Tukey tests.

**Score** **Measured area**	**Score 1 vs 0**	**p value**	**Score 2 vs 0**	**p value**	**Score 2 vs 1**	**p value**	**Score 3 vs 0**	**p value**	**Score 3 vs 1**	**p value**	**Score 3 vs 2**	**p value**
**ci**												
infiltration	2.63 (0.36–4.33)	0.0664	15.01 (10.73–20.24)	**<0.0001**	12.38 (8.19–18.08)	**0.0007**	28.81 (2.96–32.01)	**0.0028**	26.18 (0.54–29.72)	**0.0089**	13.81 (−13.21–17.2)	0.3988
fibrosis	7.65 (1.35–13.95)	**0.0098**	20.32 (10.51–30.14)	**<0.0001**	12.67 (2.35–23)	**0.0087**	44.14 (24.82–63.46)	**<0.0001**	36.49 (16.92–56.06)	**<0.0001**	23.81 (2.87–44.75)	**0.0183**
**i**												
infiltration	2.35 (0.36–4.33)	**0.0127**	15.49 (10.73–20.24)	**<0.0001**	13.14 (8.19–18.08)	**<0.0001**	17.48 (2.96–32.01)	**0.0107**	15.13 (0.54–29.72)	**0.0386**	2 (−13.21–17.2)	0.9868
fibrosis	10.58 (5.42–15.73)	**<0.0001**	26.55 (20.58–32.52)	**<0.0001**	15.97 (9.34–22.6)	**<0.0001**	42.29 (26.26–58.31)	**<0.0001**	31.71 (15.43–47.99)	**<0.0001**	15.74 (−0.8–32.28)	0.0690

Post-hoc Tukey; values are mean difference (95 % CI); bold p values are significant at p < 0.05

It has been shown that an increase in the ci score was significantly associated with an increase in the infiltration area (Table 2, Table 3, Fig. 3A,C): ci score 0 – 3.82 % (95 % CI, 2.43–5.21 %); ci score 1 – 6.46 % (95 % CI, 4.27–8.64 %, p value [ci 1 vs ci 0]=0.0664); ci score 2 – 18.83 % (95 % CI, 12.61–25.05 %, p value [ci 2 vs ci 1]=0.0007); ci score 3 – 32.64 % (95 % CI, 16.43–48.85 %, p value [ci 3 vs ci 2]=0.3988). An increase in ci score was also associated with a larger fibrosis area (Table 2, Table 3, Fig. 3B,C): ci score 0 – 16.54 % (95 % CI, 13.51–19.57 %); ci score 1 – 24.19 % (95 % CI, 20.32–28.06 %, p value [ci 1 vs ci 0]=0.0098); ci score 2 – 36.86 % (95 % CI, 29.97–43.75 %, p value [ci 2 vs ci 1]=0.0087); ci score 3 – 60.67 % (95 % CI, 46.27–75.08 %, p value [ci 3 vs ci 2]=0.0183).

The ROC analysis (Fig. 3D) showed high ability for predicting ci score 0 and 1 vs 2 and 3 based on measured parameters of infiltration area (AUC = 0.889, Sensitivity = 90.48 %, Specificity = 77.53 %, Youden’s *J* = 5.02 %) and fibrosis area (AUC = 0.8669, Sensitivity = 80.95 %, Specificity = 83.15 %, Youden’s *J* = 29.19 %).

There was a significant relationship between i score and infiltration area (ANOVA, df=3, F=38.41, p < 0.0001, Table 2, Table 3, Fig. 4A) as well as between i score and fibrosis area (ANOVA, df=3, F=46.82, p < 0.0001, Table 2, Table 3, Fig. 4B).

Increase in the i score was significantly associated with an increase in the infiltration area (Table 2, Table 3, Fig. 4A,C): i score 0 – 2.09 % (95 % CI, 1.24–2.95 %); i score 1 – 4.44 % (95 % CI, 2.87–6.01 %, p value [i 1 vs i 0]=0.0127); i score 2 – 17.58 % (95 % CI, 13.91–21.25 %, p value [i 2 vs i 1]<0.0001); i score 3 – 19.57 % (95 % CI, 8.48–30.67 %, p value [i 3 vs i 2]=0.9868). An increase in i score was also associated with a larger fibrosis area (Table 2, Table 3, Fig. 4B,C): i score 0 – 10.98 % (95 % CI, 8.61–13.35 %); i score 1 – 21.55 % (95 % CI, 18.33–24.78 %, p value [i 1 vs i 0] <0.0001); i score 2 – 37.52 % (95 % CI,33.6–41.45 %, p value [i 2 vs i 1]<0.0001); i score 3 – 53.26 % (95 % CI, 41.27–65.26 %, p value [i 3 vs i 2]=0.0690).

The ROC analysis (Fig. 4D) showed high ability for predicting i score 0 and 1 vs 2 and 3 based on measured parameters of infiltration area (AUC = 0.9263, Sensitivity = 94.29 %, Specificity = 78.67 %, Youden’s *J* = 3.04 %) and fibrosis area (AUC = 0.8971, Sensitivity = 71.43 %, Specificity = 94.67 %, Youden’s *J* = 29.55 %).

To assess the model's performance, we compared its accuracy on WSIs of renal parenchyma with expert annotations from an external dataset. The Dice scores for the different classes of annotations were as follows: “tubuli”: 0.8612, “glomeruli”: 0.9567, “arteries”: 0.7036 and background (“stroma”): 0.7033. Comparing the Dice scores between the internal and external datasets, it is evident that our model performed consistently well across different datasets, with slightly higher Dice scores observed in the internal dataset for tissue structure annotations.

Figure A in Appendices demonstrates the efficacy of our model in accurately segmenting structures in kidney tissue. The comparison between the ground truth and predictions shows a high degree of accuracy, with the model closely matching the pathologist's annotations. Minor discrepancies are noted but do not significantly detract from the algorithm’s overall performance. Notably, the algorithm accurately identified modified structures, including tubules showing signs of dystrophy and vessels with slight hyalinosis. This visual validation supports the quantitative metrics, such as Dice scores, in assessing the model's effectiveness. Additionally, the confusion matrix in Figure B illustrates our model's performance in distinguishing between the different classes. The comparison with confusion matrix of the internal dataset reveals that algorithm performed robustly in different datasets. Detection of "glomeruli" is consistently high, while others classes show moderate variability.

## 4 Discussion

This study is the first to report a CV model that not only identifies tissue structures and predicts prognosis but also provides quantitative alternatives to the two most impactful metrics—fibrosis and interstitial infiltration—essential for diagnosis within the Banff classification system. These morphological findings are crucial in differentiating between borderline changes and rejection. Our study introduces a deep learning model that significantly enhances the objectivity and efficiency of diagnosing acute kidney transplant rejection. This AI solution benefits clinicians by demonstrating that automation can address the issue of interobserver variability, potentially leading to more personalized treatments and improved patient outcomes. Additionally, our work illustrates that combination of neural networks with traditional machine learning models can provide complex analysis of both tissue and cellular components of biological tissues. The present research bridges the gap between established clinical system and the data-driven analysis, demonstrating the necessity for such models to transform current clinical practices.

Nephropathology AI studies stand out among all digital pathology research due to the focus on non-malignant tissues. Repeated percutaneous biopsies of kidney transplants and whole-organ removal of kidneys with renal cancer allowed to accumulate vastnumbers of histological slides required to study normal tissue composition. In a paper by Boutelja et al., convolutional neural networks were trained for the segmentation of healthy rat renal parenchyma and further applied to evaluate patient samples with diseases (minimal change disease, acute tubular injury, and glomerulonephritis). Based on approximately 72,700 annotations, six primary kidney structures were identified: glomeruli, glomerular tufts, tubules, veins, arteries and their lumina. The model was used to evaluate interstitial expansion, tubular dilation and atrophy and glomerular size variability [15]. In an earlier article by Hermsen et al., a neural network was trained for segmentation of 11 classes of tissue structures in kidney transplant biopsies. The calculations were used to predict several Banff system scores (ci, ti, ct), and a correlation was observed between histologist evaluation and the convolutional neural network [12]. Our work is also based on identifying kidney tissue structures and abnormalities in WSI, however, the focus on interstitial infiltration let us make more valuable prognostic evaluations for acute renal graft rejection.

More recently, AI was used for differentiating normal and pathological structures. The image processing methods based on machine learning can differentiate normal and sclerosed glomeruli with an accuracy of up to 0.98 [9]. A significant advancement is the application of AI for the diagnosis of various kidney diseases. Machine learning was employed to divide kidney biopsy samples into two groups: kidney tissue without changes and kidney tissue with pathological alterations. The CNN was able to diagnose kidney rejection among all pathological conditions [16]. These findings closed the gap between fundamental science and contemporary problems in the field of nephropathology.

The present study fits in the trend of implementing AI technologies for the diagnosis of kidney transplant status. Evaluation of fibrotic changes (ci score of the Banff system) in renal graft biopsies is important since this metric correlates with the loss of kidney function. There is still a debate on the staining method that should become standard in digital pathology routine. In the study by Farris et al., interstitial fibrosis was assessed using an immunohistochemical reaction with an antibody to collagen type III and Masson's trichrome-staining kit. The authors believe that the use of additional staining methods (including immunohistochemistry) of histological preparations, rather than routinely stained hematoxylin and eosin slides, can contribute to the standardization of the process. However, it should be noted that both immuno- and histochemical staining methods need to be validated in each laboratory to achieve consistent scanning conditions. In the same publication, Aperio ImageScope Positive Pixel Count algorithm was used to calculate the percentage of tubular atrophy and cortical substance infiltration [17].

We chose hematoxylin and eosin stained histological slides in order to reach maximum clinical translatability of our model. It is worth noting that several reported AI algorithms were trained on WSI stained with special stainings (Masson's trichrome, immunohistochemistry with antibodies to collagens, CD34, CD3) which are used with variation in reagents and protocols in different laboratories or not used in the daily practice of a pathologist [8], [10], [11], [12]. On the other hand, immunohistochemistry combined with stroma segmentation is necessary for AI-assisted assessment of C4d deposits in peritubular capillaries that has been successfully applied for the detection of humoral (antibody-mediated) rejection [18].

**Fig. 5:**
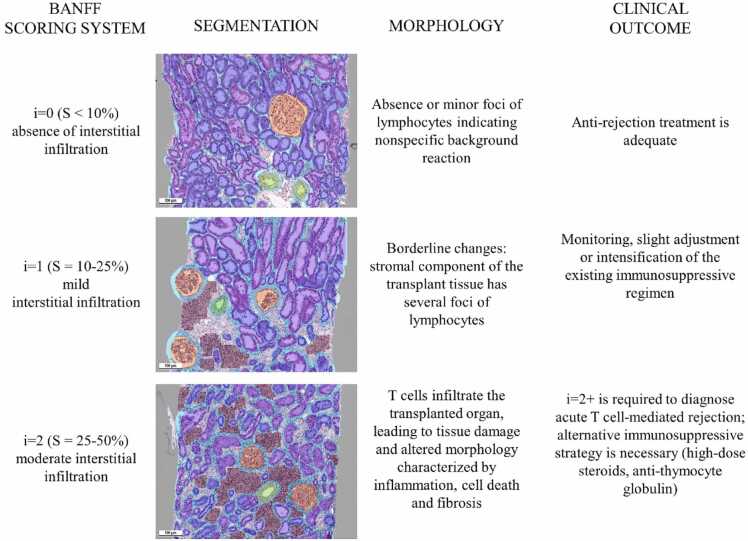
Precise evaluation of pathological changes (e.g., interstitial infiltration) is important for adequate therapeutic strategy. Artificial intelligence-based models will become indispensable tools for pathologists studying cases with borderline pathological changes through reliable calculation of areas (S) with pathological findings.

Our results strongly indicate that the AI model can detect areas of immune cell infiltration and predict the relevant clinical (i) score with a high precision. We base our statement on prediction of the expert scores on ROC-analysis, sensitivity and specificity assessment. This measurement tool can be of use to pathologists when there are doubts in distinguishing i = 1 and i = 2. This minor score difference defines the therapeutic strategy and can become a question of life and death for the patient (Fig. 5). Also, our model allowed pathologists to select specific regions without fibrosis or edema for accurate infiltration measurement. However, we used the entire renal cortex for statistical analysis, which still accurately predicted expert scores in our samples. The metrics we obtained correlated strongly with the experts' assessment of biopsies according to the Banff classification. Using our model, it is possible to accurately predict the percentage of i-score, rather than an approximation as with traditional light microscopy. We can also identify clear class boundaries in the i-score based on the percentages estimated by our model, which can be used to further refine the Banff classification. To help the pathologist make the right decision, AI was applied to determine the severity of inflammatory infiltration in the interstitial tissue of kidney biopsies after transplantation. Immune cells of various types (T-killers, T-helpers, B-lymphocytes, macrophages) were evaluated based on parameters such as the nucleus-to-cytoplasm ratio, cell density, cell-to-cell ratio and microenvironment. However, the study was conducted on immunohistochemical preparations, which significantly complicates and slows down the transition of the approach to clinical practice [19].

In the publication by Moon A. et al., interstitial infiltration was assessed using built-in methods in the Aperio scanner software, as well as a customizable algorithm with open-source code, and was applied to specimens stained with antibodies against CD3. Cellular density per 1 mm^2^ was calculated. This method can be considered widely reproducible in various laboratories equipped with the necessary tools, but a more translatable decision can be designed for a range of scanners [11].

We chose to develop AI model that works with WSI rather than in the histological scanner operating system as a step in the direction of multi-module pathomorphological ecosystem. On the other hand, many publications reported valuable results that were obtained in manufacturer’s morphometric software without programmed tissue specificity, such as the Aperio Positive Pixel Count Algorithm (Leica Biosystems) or the Aperio microvessel density Algorithm (Leica Biosystems) [8], [10], [11], [12].

Recently, a pipeline for predicting early and long-term graft survival was proposed by Yi et al. It is based on the evaluation of pathological changes in initial biopsy and kidney transplant biopsy one year later. Abnormalities in interstitium and tubules and immune cell infiltrated regions were assessed in the biopsies according to the 2007 Banff classification. This method represents a novel approach for risk stratification of allografts and post-transplant monitoring in clinical practice [20].

The machine learning is being implemented in the routine of morphological assessment of kidney samples. Ligabue et al. employed a convolutional neural network to immunofluorescence study of kidney biopsiesand identified different types of immunoglobulins, kappa and lambda chains, as well as complement system components deposited in the glomerular capillaries. The researchers noted that the use of AI as a decision support system for pathologists accelerates the biopsy assessment process by tens of times [21]. A new close frontier for kidney structure segmentation can be in the area of 3D histological models, or biopsy duplicates [22]. It can be imagined how the value of morphological analysis can increase from addition of a third dimension since we can compare this to a transition from conventional radiography to X-ray computer tomography. We envision automated morphometric evaluation of glomerular tuft and tubular shapes that can compete with highly complicated methods such as electron microscopy in diagnostics of renal pathology.

One of the limitations of the program was that its work was evaluated only in a manually selected cortical part of the renal biopsy. The integration of the program in the medical practice would require a semantic segmentation of renal tissue compartments. We believe that this problem can be solved by applying such neural networks as transformers [23], [24]. However, it will require a significant computational resources and was out of the scope of the present work. Another limitation of this project was that immune cell detection was not precise inside tubules and arteries which could assist in evaluation of tubulitis and arteritis. The proposed approach had a low specificity due to mistaken detection of tubular and blood vessel cell nuclei as immune cells. We intend to train an original classifier for tubules and arteries with signs of tubulitis and arteritis which would be beneficial in graft evaluation, glomerulonephritis and acute tubular necrosis. A major limitation in relation to clinical translation would be that small sizes of the tissue sections and processing artefacts resulted in several outlying values (especially for fibrosis). Those were also the histological slides that were prepared as long as 20 years ago. Implementing quality control in digital pathology departments will increase the model precision in medical practice. The model generalizability is currently limited by the diversity of slide preparation methods and the singular scanning device used. To improve generalizability, we added an external cohort in our investigation. Future research should aim to validate our findings in a broader, multi-center context to enhance the reliability and applicability of the AI model in diverse clinical settings. In the future research we intend to use a combination of tiles of different sizes or achieve accurate delineation of tissue structures and alleviate the patching artifacts. Furthermore, our deep learning model is specifically trained to detect fibrosis and interstitial infiltration, which are characteristic of cell-mediated rejection in renal transplants. In our future work, we plan to enhance our algorithm to identify additional markers of cell-mediated rejection, such as tubulitis, as well as indicators of antibody-mediated rejection, including peritubular capillaritis, glomerulitis and C4d deposition. Automated quantitative analysis of tubulitis (t-scoring) would be a valuable tool in analyzing diverse range of pathologies, including tubulointerstitial nephritis, various types of rejection and viral graft lesions. In this study, we demonstrated how AI segmentation can serve as an alternative to expert estimation of the relative area of histological findings. However, it appears that automated t-scoring remains an unresolved problem primarily associated with image classification between normal tubuli, dystrophic tubuli and tubuli with tubulitis. This challenge extends beyond the automation of Banff classification and requires a fundamentally different approach. This would provide a more comprehensive tool for diagnosing various types of renal transplant rejection and improve patient outcomes.The integration of artificial intelligence in nephropathology has demonstrated significant potential for the diagnosis, prognosis and management of kidney transplant rejection and other renal diseases. The use of AI-driven models in evaluating immune cell infiltration, fibrotic changes and various pathological findings has shown promising results in terms of accuracy and efficiency, helping pathologists in their decision-making process. Further evolution of feature extraction from WSIs and development of efficient computational methods for histological analysis pave a path for a data-driven diagnostics [25], [26].

## 5 Conclusions

As technology continues to advance, AI-assisted analysis of kidney biopsies and WSI may become an essential part of the pathomorphological ecosystem, enabling more accurate diagnoses and personalized treatment plans for patients. Future research should focus on validating these AI algorithms in different laboratories, developing standardization processes and exploring the potential of 3D histological models to further enhance the value of morphological analysis in nephropathology.

## Funding

This work was supported by the 10.13039/501100006769Russian Science Foundation (Grant No. 22-15-00467).

## CRediT authorship contribution statement

**Alexander Arutyunyan:** Writing – review & editing, Resources, Project administration, Methodology. **Alina Kalinichenko:** Writing – review & editing, Validation, Investigation. **Elena Ivanova:** Writing – original draft, Validation, Software, Investigation. **Aleksey Lychagin:** Writing – review & editing, Software, Formal analysis. **Alexey Fayzullin:** Writing – original draft, Visualization, Project administration, Methodology, Investigation, Formal analysis, Data curation, Conceptualization. **Alesia Bakulina:** Writing – review & editing, Validation, Investigation. **Maxim Balyasin:** Writing – original draft, Visualization, Software, Methodology, Investigation, Formal analysis. **Yana Valieva:** Writing – review & editing, Investigation. **Pavel Nikitin:** Writing – review & editing, Validation, Investigation. **Victor Grinin:** Writing – review & editing, Visualization, Software, Methodology, Investigation, Formal analysis. **Peter Timashev:** Writing – review & editing, Supervision, Resources, Project administration, Investigation, Funding acquisition, Conceptualization. **Svetlana Solovyeva:** Writing – review & editing, Validation, Investigation. **Dmitry Ermilov:** Writing – original draft, Software, Resources, Project administration, Data curation.

## Declaration of Competing Interest

The authors declare the following financial interests/personal relationships which may be considered as potential competing interests: Alexander Arutyunyan reports a relationship with PJSC VimpelCom that includes: employment. Victor Grinin reports a relationship with PJSC VimpelCom that includes: employment. Dmitry Ermilov reports a relationship with PJSC VimpelCom that includes: employment. If there are other authors, they declare that they have no known competing financial interests or personal relationships that could have appeared to influence the work reported in this paper.

## Figures and Tables

**Figure:**
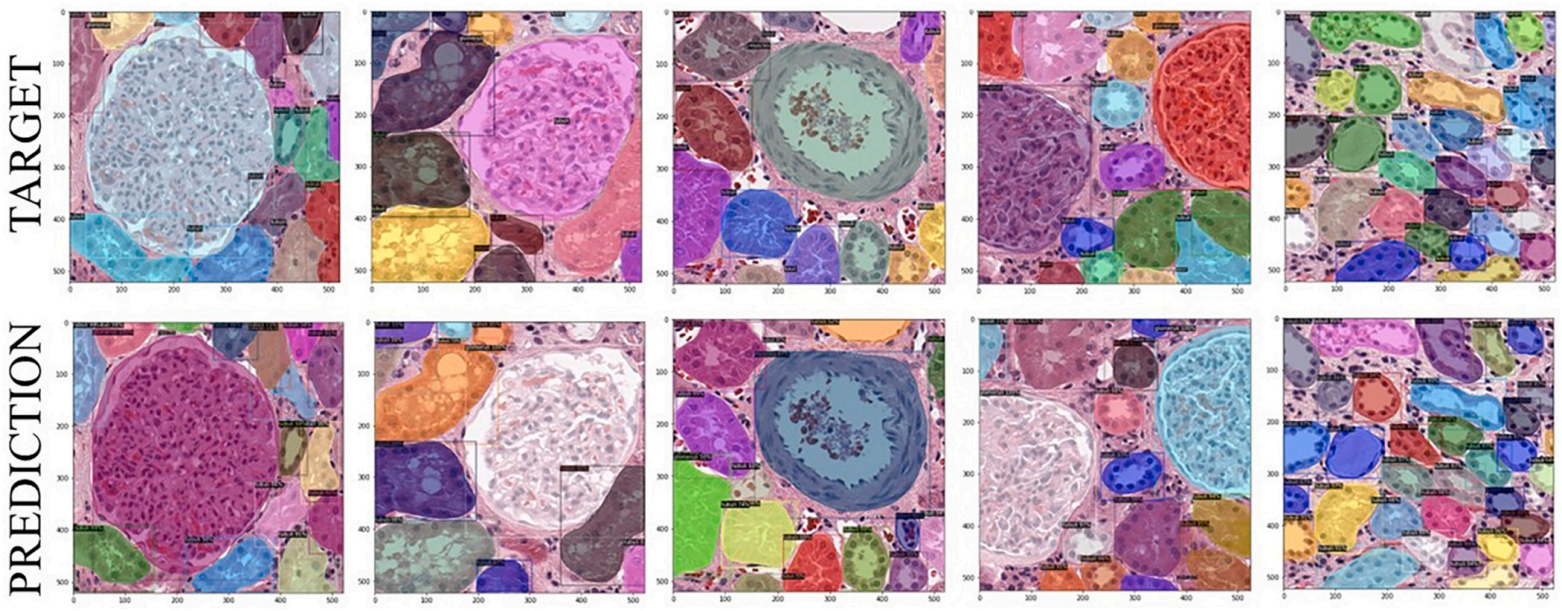


**Figure:**
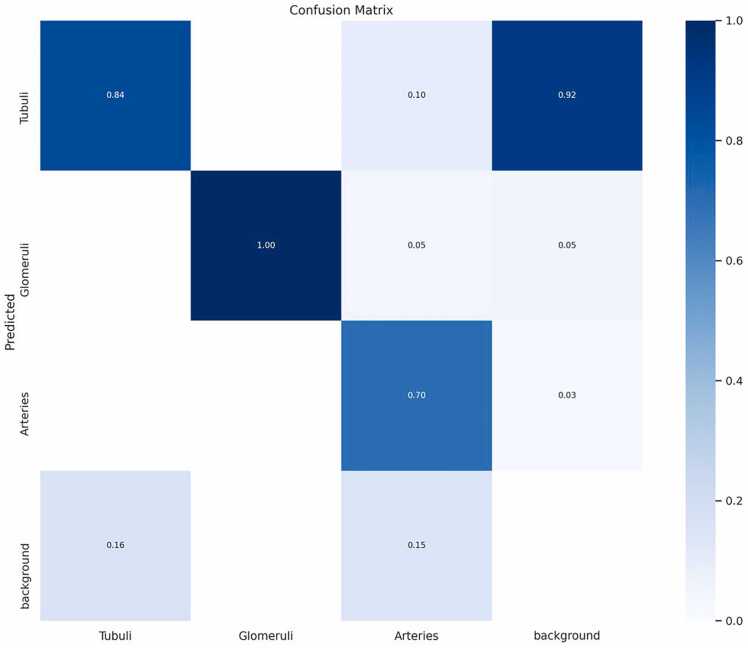


## Data Availability

The datasets generated during and/or analyzed during the current study are available from the corresponding author on reasonable request. In Supplementary Material we added compressed file contains a QuPath project and a dataset with images from an external cohort, which were used in our study.

## References

[1] Wolfe R.A., Ashby V.B., Milford E.L., Ojo A.O., Ettenger R.E., Agodoa L.Y., Held P.J., Port F.K. (1999). Comparison of mortality in all patients on dialysis, patients on dialysis awaiting transplantation, and recipients of a first cadaveric transplant. N Engl J Med.

[2] Hatzinger M., Stastny M., Grutzmacher P., Sohn M. (2016). Die Geschichte der Nierentransplantation [The history of kidney transplantation]. Urologe.

[3] Saran R., Robinson B., Abbott K.C., Agodoa L.Y.C., Bragg-Gresham J., Balkrishnan R., Bhave N., Dietrich X., Ding Z., Eggers P.W., Gaipov A., Gillen D., Gipson D., Gu H., Guro P., Haggerty D., Han Y., He K., Herman W., Heung M., Hirth R.A., Hsiung J.T., Hutton D., Inoue A., Jacobsen S.J., Jin Y., Kalantar-Zadeh K., Kapke A., Kleine C.E., Kovesdy C.P., Krueter W., Kurtz V., Li Y., Liu S., Marroquin M.V., McCullough K., Molnar M.Z., Modi Z., Montez-Rath M., Moradi H., Morgenstern H., Mukhopadhyay P., Nallamothu B., Nguyen D.V., Norris K.C., O'Hare A.M., Obi Y., Park C., Pearson J., Pisoni R., Potukuchi P.K., Repeck K., Rhee C.M., Schaubel D.E., Schrager J., Selewski D.T., Shamraj R., Shaw S.F., Shi J.M., Shieu M., Sim J.J., Soohoo M., Steffick D., Streja E., Sumida K., Kurella Tamura M., Tilea A., Turf M., Wang D., Weng W., Woodside K.J., Wyncott A., Xiang J., Xin X., Yin M., You A.S., Zhang X., Zhou H., Shahinian V. (2019). US Renal Data System 2018 annual data report: epidemiology of kidney disease in the United States. Am J Kidney Dis.

[4] Jeong H.J. (2020). Diagnosis of renal transplant rejection: Banff classification and beyond. Kidney Res Clin Pr.

[5] Hart A., Lentine K.L., Smith J.M., Miller J.M., Skeans M.A., Prentice M., Robinson A., Foutz J., Booker S.E., Israni A.K., Hirose R., Snyder J.J. (2021). OPTN/SRTR 2019 annual data report: kidney. Am J Transpl.

[6] Roufosse C., Simmonds N., Clahsen-van Groningen M., Haas M., Henriksen K.J., Horsfield C., Loupy A., Mengel M., Perkowska-Ptasińska A., Rabant M., Racusen L.C., Solez K., Becker J.U. (2018). A 2018 reference guide to the banff classification of renal allograft pathology. Transplantation.

[7] Loupy A., Mengel M., Haas M. (2022). Thirty years of the International Banff Classification for Allograft Pathology: the past, present, and future of kidney transplant diagnostics. Kidney Int.

[8] Farris A.B., Ellis C.L., Rogers T.E., Lawson D., Cohen C., Rosen S. (2016). Renal medullary and cortical correlates in fibrosis, epithelial mass, microvascularity, and microanatomy using whole slide image analysis morphometry. PLoS One.

[9] Cascarano G.D., Debitonto F.S., Lemma R., Brunetti A., Buongiorno D., De Feudis I., Guerriero A., Venere U., Matino S., Rocchetti M.T., Rossini M., Pesce F., Gesualdo L., Bevilacqua V. (2021). A neural network for glomerulus classification based on histological images of kidney biopsy. BMC Med Inf Decis Mak.

[10] Nicholson M.L., Bailey E., Williams S., Harris K.P., Furness P.N. (1999). Computerized histomorphometric assessment of protocol renal transplant biopsy specimens for surrogate markers of chronic rejection. Transplantation.

[11] Moon A., Smith G.H., Kong J., Rogers T.E., Ellis C.L., Farris A.B.B. (2018). Development of CD3 cell quantitation algorithms for renal allograft biopsy rejection assessment utilizing open source image analysis software. Virchows Arch.

[12] Hermsen M., de Bel T., den Boer M., Steenbergen E.J., Kers J., Florquin S., Roelofs J.J.T.H., Stegall M.D., Alexander M.P., Smith B.H., Smeets B., Hilbrands L.B., van der Laak J.A.W.M. (2019). Deep learning-based histopathologic assessment of kidney tissue. J Am Soc Nephrol.

[13] Wu Y., Kirillov A., Massa F., Lo W.-Y., Girshick R. (2019) Detectron2. https://github.com/facebookresearch/detectron2.

[bib14] 14National Cancer Institute Clinical Proteomic Tumor Analysis Consortium (CPTAC). (2018). The Clinical Proteomic Tumor Analysis Consortium Clear Cell Renal Cell Carcinoma Collection (CPTAC-CCRCC) (Version 13) [Data set]. The Cancer Imaging Archive. https://doi.org/10.7937/k9/tcia.2018.oblamn27

[15] Bouteldja N., Klinkhammer B.M., Bülow R.D., Droste P., Otten S.W., Freifrau von Stillfried S., Moellmann J., Sheehan S.M., Korstanje R., Menzel S., Bankhead P., Mietsch M., Drummer C., Lehrke M., Kramann R., Floege J., Boor P., Merhof D. (2021). Deep learning-based segmentation and quantification in experimental kidney histopathology. J Am Soc Nephrol.

[16] Kers J., Bülow R.D., Klinkhammer B.M., Breimer G.E., Fontana F., Abiola A.A., Hofstraat R., Corthals G.L., Peters-Sengers H., Djudjaj S., von Stillfried S., Hölscher D.L., Pieters T.T., van Zuilen A.D., Bemelman F.J., Nurmohamed A.S., Naesens M., Roelofs J.J.T.H., Florquin S., Floege J., Nguyen T.Q., Kather J.N., Boor P. (2022). Deep learning-based classification of kidney transplant pathology: a retrospective, multicentre, proof-of-concept study. Lancet Digit Health.

[17] Farris A.B., Chan S., Climenhaga J., Adam B., Bellamy C.O., Seron D., Colvin R.B., Reeve J., Mengel M. (2014). Banff fibrosis study: multicenter visual assessment and computerized analysis of interstitial fibrosis in kidney biopsies. Am J Transpl.

[18] Kim Y.G., Choi G., Go H., Cho Y., Lee H., Lee A.R., Park B., Kim N. (2019). A fully automated system using a convolutional neural network to predict renal allograft rejection: extra-validation with giga-pixel immunostained slides. Sci Rep.

[19] Hermsen M., Volk V., Bräsen J.H., Geijs D.J., Gwinner W., Kers J., Linmans J., Schaadt N.S., Schmitz J., Steenbergen E.J., Swiderska-Chadaj Z., Smeets B., Hilbrands L.B., Feuerhake F., van der Laak J.A.W.M. (2021). Quantitative assessment of inflammatory infiltrates in kidney transplant biopsies using multiplex tyramide signal amplification and deep learning. Lab Investig.

[20] Yi Z., Salem F., Menon M.C., Keung K., Xi C., Hultin S., Haroon Al Rasheed M.R., Li L., Su F., Sun Z., Wei C., Huang W., Fredericks S., Lin Q., Banu K., Wong G., Rogers N.M., Farouk S., Cravedi P., Shingde M., Smith R.N., Rosales I.A., O'Connell P.J., Colvin R.B., Murphy B., Zhang W. (2022). Deep learning identified pathological abnormalities predictive of graft loss in kidney transplant biopsies. Kidney Int.

[21] Ligabue G., Pollastri F., Fontana F., Leonelli M., Furci L., Giovanella S., Alfano G., Cappelli G., Testa F., Bolelli F., Grana C., Magistroni R. (2020). Evaluation of the classification accuracy of the kidney biopsy direct immunofluorescence through convolutional neural networks. Clin J Am Soc Nephrol.

[22] Kiemen A.L., Braxton A.M., Grahn M.P., Han K.S., Babu J.M., Reichel R., Jiang A.C., Kim B., Hsu J., Amoa F. (2022). CODA: quantitative 3D reconstruction of large tissues at cellular resolution. Nat Methods.

[23] Gadermayr M., Gupta L., Appel V., Boor P., Klinkhammer B.M., Merhof D. (2019). Generative adversarial networks for facilitating stain-independent supervised and unsupervised segmentation: a study on kidney histology. IEEE Trans Med Imaging.

[24] Cong C., Liu S., Di Ieva A., Pagnucco M., Berkovsky S., Song Y. (2022). Colour adaptive generative networks for stain normalisation of histopathology images. Med Image Anal.

[25] Hölscher D.L., Bouteldja N., Joodaki M., Russo M.L., Lan Y.-C., Sadr A.V., Cheng M., Tesar V., Stillfried S.V., Klinkhammer B. (2023). Next-generation morphometry for pathomics-data mining in histopathology. Nat Commun.

[26] Ivanova E., Fayzullin A., Grinin V., Ermilov D., Arutyunyan A., Timashev P., Shekhter A. (2023). Empowering renal cancer management with AI and digital pathology: pathology, diagnostics and prognosis. Biomedicines.

