# Supplementary figures and images for: Towards accurate and efficient diagnoses in nephropathology: An AI-based approach for assessing kidney transplant rejection
Source: Comput Struct Biotechnol J. 2024 Aug 16;24:571–82. doi: 10.1016/j.csbj.2024.08.011 (PMC11385065; doi:10.1016/j.csbj.2024.08.011)

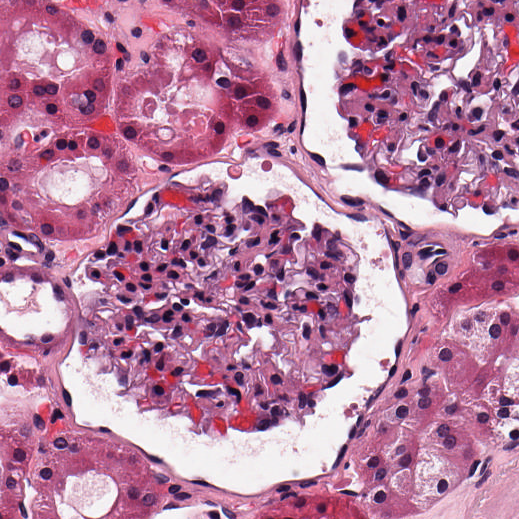

Supplement: Supplementary file 2 — Supplementary material [file mmc1.zip › verification/C3L-00004-26 (1, x=8752, y=30970, w=519, h=519).png]

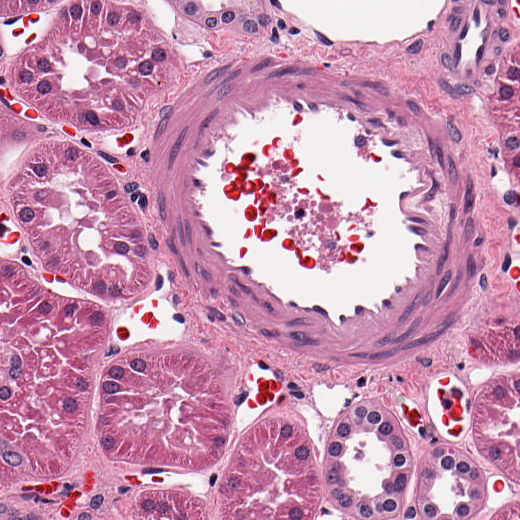

Supplement: Supplementary file 2 — Supplementary material [file mmc1.zip › verification/C3L-00010-26 (1, x=13233, y=17218, w=520, h=520).png]

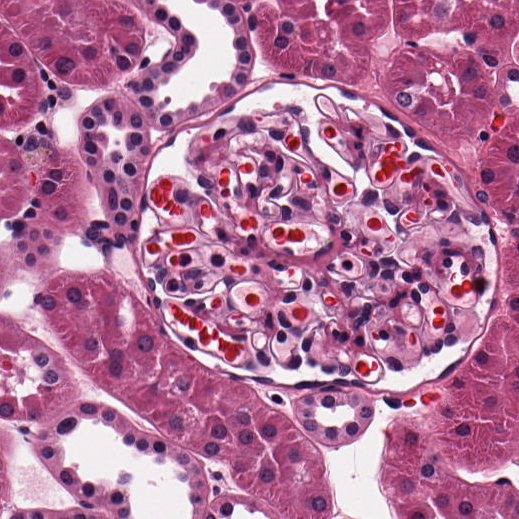

Supplement: Supplementary file 2 — Supplementary material [file mmc1.zip › verification/C3L-00011-26 (1, x=5878, y=8241, w=519, h=519).png]

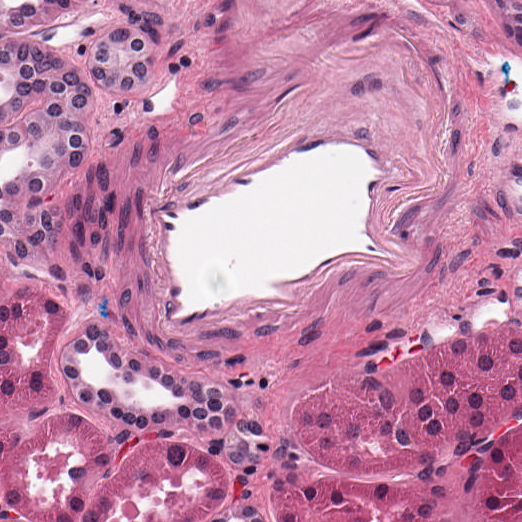

Supplement: Supplementary file 2 — Supplementary material [file mmc1.zip › verification/C3L-00026-26 (1, x=11740, y=16115, w=522, h=522).png]

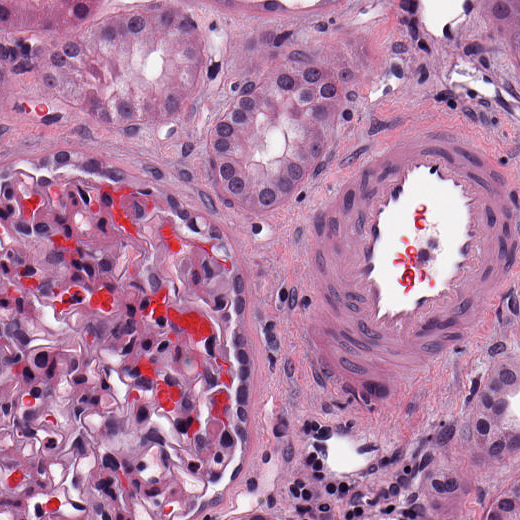

Supplement: Supplementary file 2 — Supplementary material [file mmc1.zip › verification/C3L-00079-26 (1, x=10645, y=6048, w=520, h=520).png]

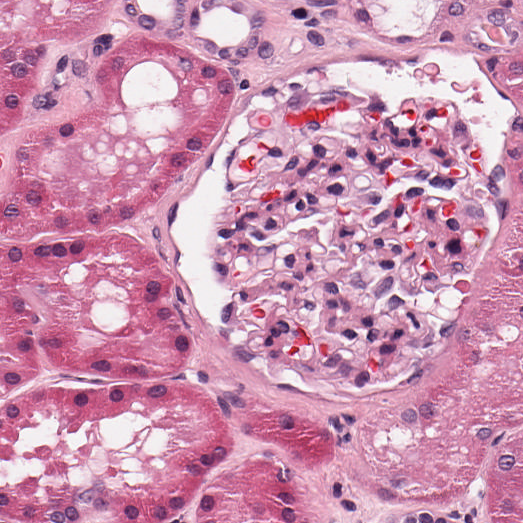

Supplement: Supplementary file 2 — Supplementary material [file mmc1.zip › verification/C3L-00088-26 (1, x=9632, y=16270, w=523, h=523).png]

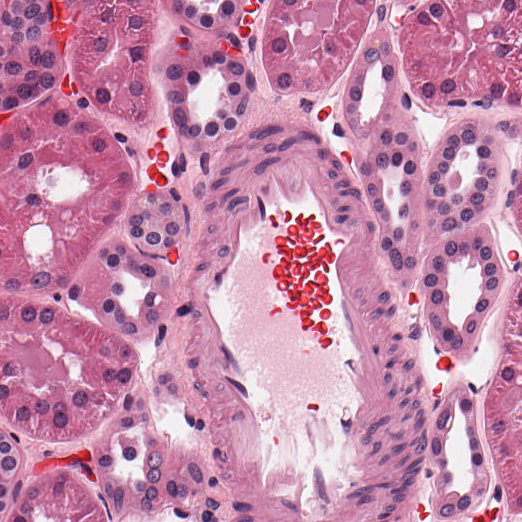

Supplement: Supplementary file 2 — Supplementary material [file mmc1.zip › verification/C3L-00097-26 (1, x=9001, y=12938, w=522, h=522).png]

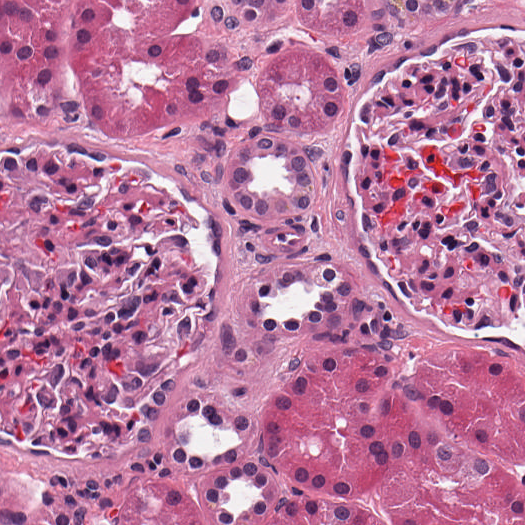

Supplement: Supplementary file 2 — Supplementary material [file mmc1.zip › verification/C3L-00103-26 (1, x=5987, y=14953, w=525, h=525).png]

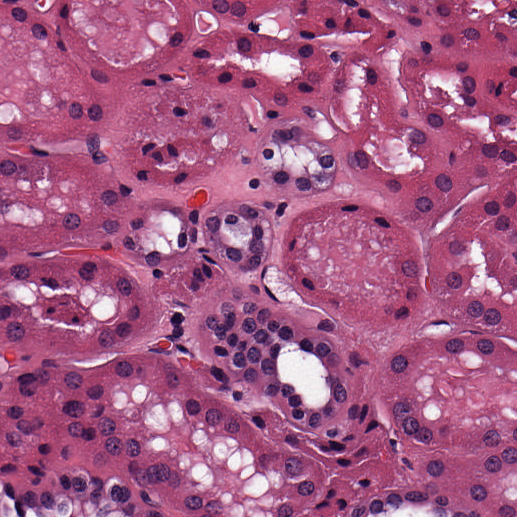

Supplement: Supplementary file 2 — Supplementary material [file mmc1.zip › verification/C3L-00183-26 (1, x=6151, y=3859, w=517, h=517).png]

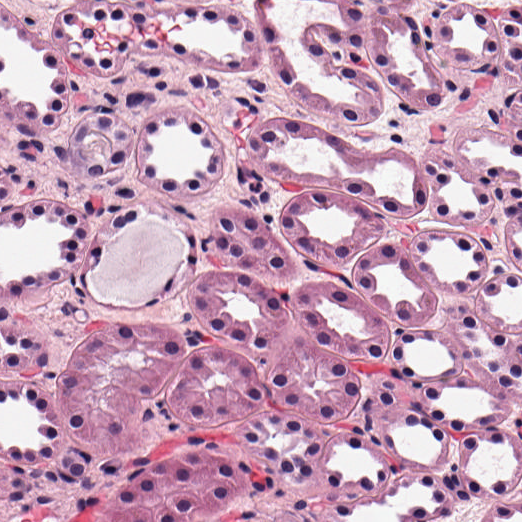

Supplement: Supplementary file 2 — Supplementary material [file mmc1.zip › verification/C3L-00359-26 (1, x=15820, y=3291, w=522, h=522).png]

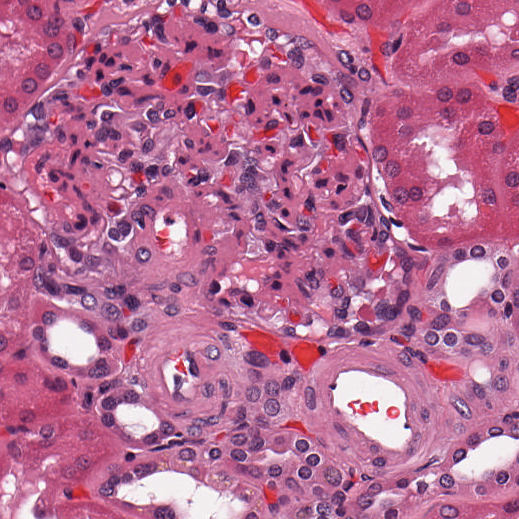

Supplement: Supplementary file 2 — Supplementary material [file mmc1.zip › verification/C3L-00360-26 (1, x=6029, y=19777, w=519, h=519).png]

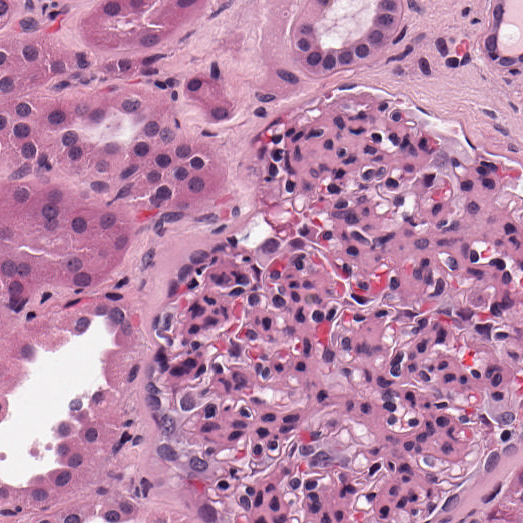

Supplement: Supplementary file 2 — Supplementary material [file mmc1.zip › verification/C3L-00416-26 (1, x=7755, y=8272, w=523, h=523).png]

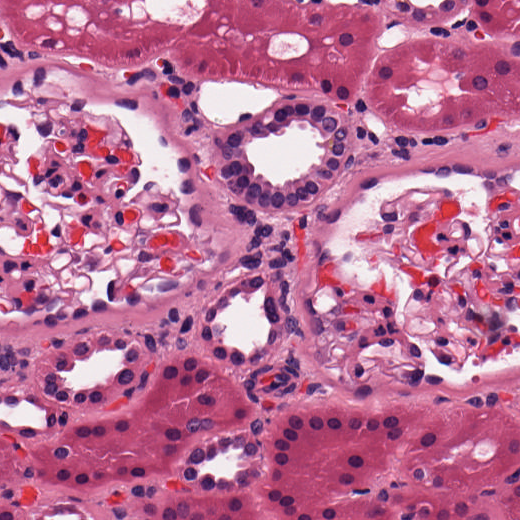

Supplement: Supplementary file 2 — Supplementary material [file mmc1.zip › verification/C3L-00418-26 (1, x=11783, y=12095, w=520, h=520).png]

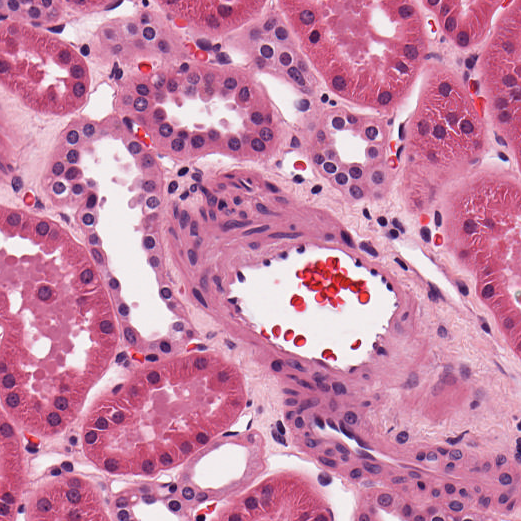

Supplement: Supplementary file 2 — Supplementary material [file mmc1.zip › verification/C3L-00447-26 (1, x=8123, y=18877, w=521, h=521).png]

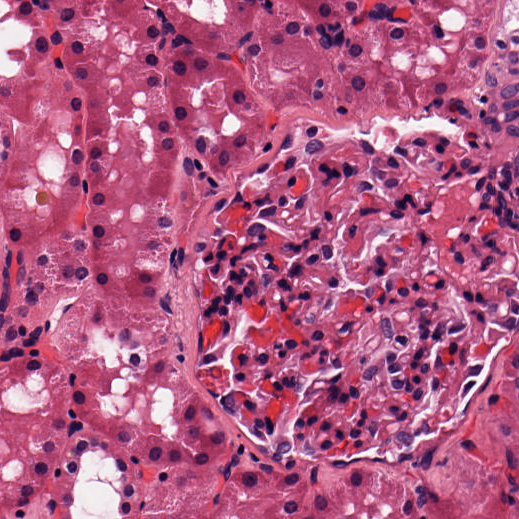

Supplement: Supplementary file 2 — Supplementary material [file mmc1.zip › verification/C3L-00448-26 (1, x=17437, y=16624, w=519, h=519).png]

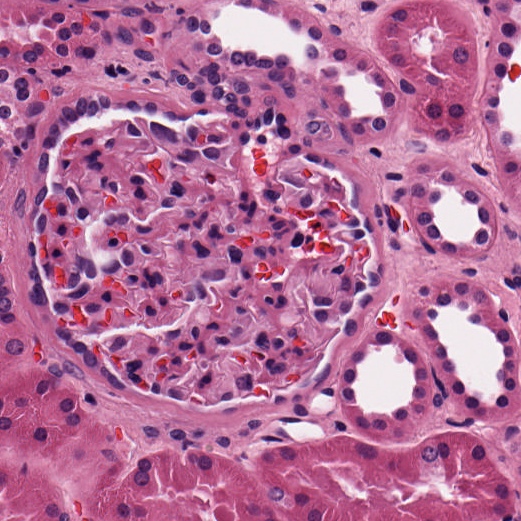

Supplement: Supplementary file 2 — Supplementary material [file mmc1.zip › verification/C3L-00561-26 (1, x=11543, y=21813, w=521, h=521).png]

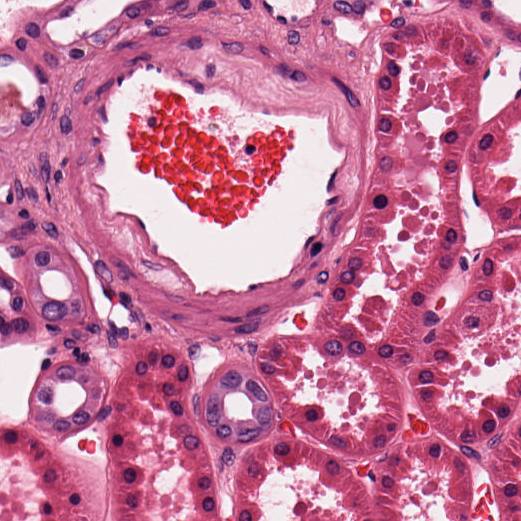

Supplement: Supplementary file 2 — Supplementary material [file mmc1.zip › verification/C3L-00581-26 (1, x=15938, y=10526, w=521, h=521).png]

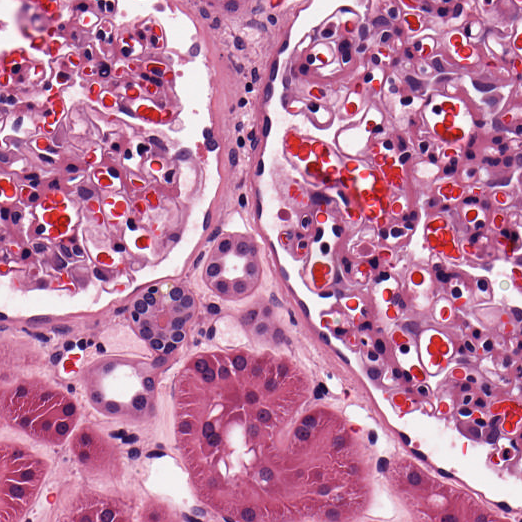

Supplement: Supplementary file 2 — Supplementary material [file mmc1.zip › verification/C3L-00583-26 (1, x=14523, y=18290, w=522, h=522).png]

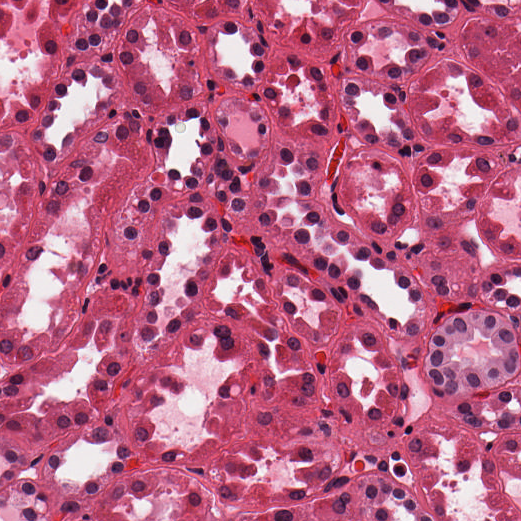

Supplement: Supplementary file 2 — Supplementary material [file mmc1.zip › verification/C3L-00606-26 (1, x=4781, y=18059, w=521, h=521).png]

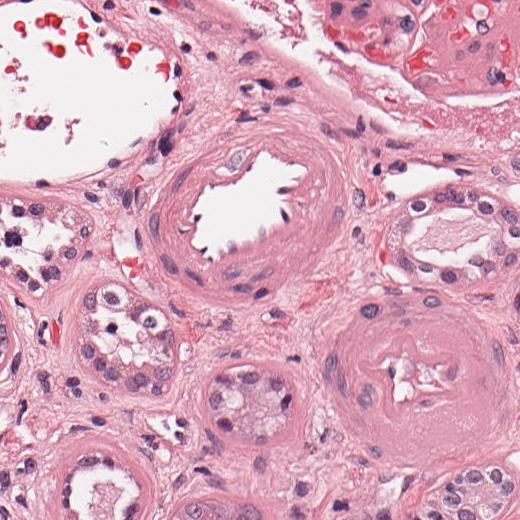

Supplement: Supplementary file 2 — Supplementary material [file mmc1.zip › verification/C3L-00607-22 (1, x=11613, y=10842, w=520, h=520).png]

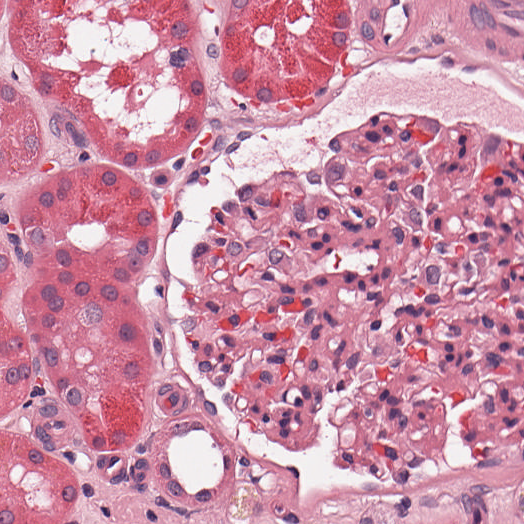

Supplement: Supplementary file 2 — Supplementary material [file mmc1.zip › verification/C3L-00791-22 (1, x=9469, y=6752, w=524, h=524).png]

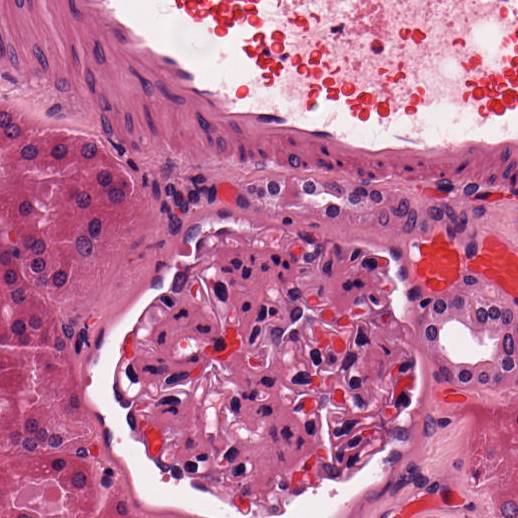

Supplement: Supplementary file 2 — Supplementary material [file mmc1.zip › verification/C3L-00907-26 (1, x=20374, y=22574, w=518, h=518).png]

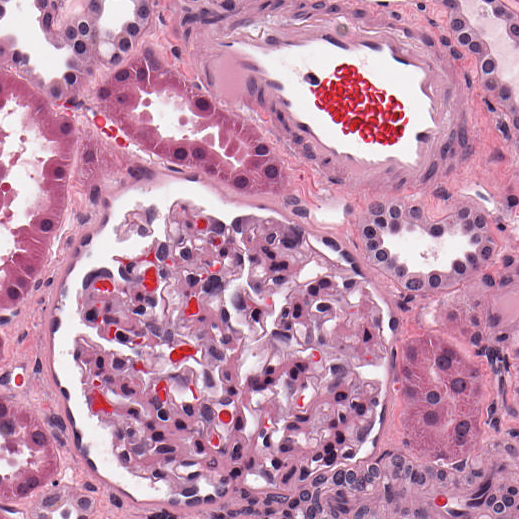

Supplement: Supplementary file 2 — Supplementary material [file mmc1.zip › verification/C3L-00908-26 (1, x=12934, y=16524, w=519, h=519).png]

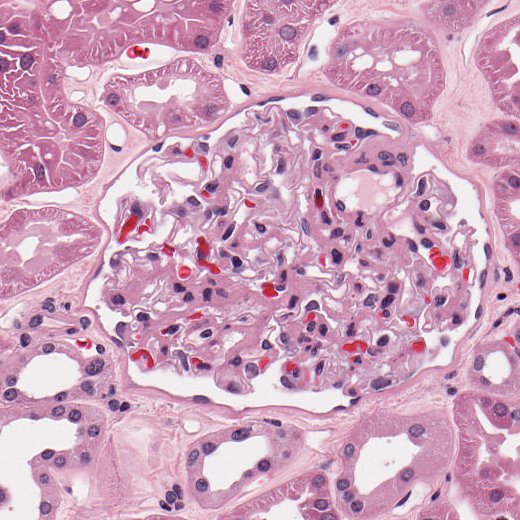

Supplement: Supplementary file 2 — Supplementary material [file mmc1.zip › verification/C3L-00910-26 (1, x=12307, y=29319, w=520, h=520).png]

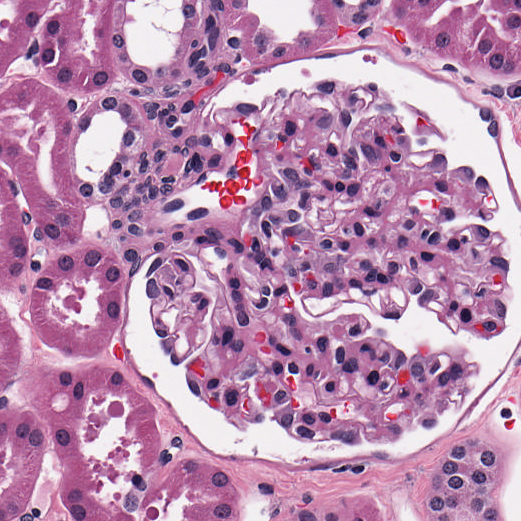

Supplement: Supplementary file 2 — Supplementary material [file mmc1.zip › verification/C3L-00966-26 (1, x=18159, y=8258, w=521, h=521).png]

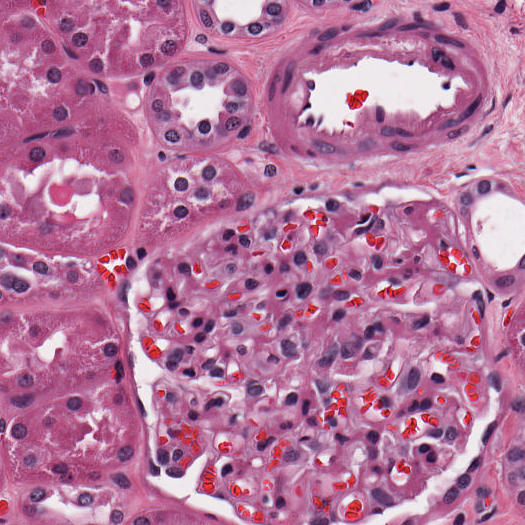

Supplement: Supplementary file 2 — Supplementary material [file mmc1.zip › verification/C3L-00968-27 (1, x=16668, y=11463, w=525, h=525).png]

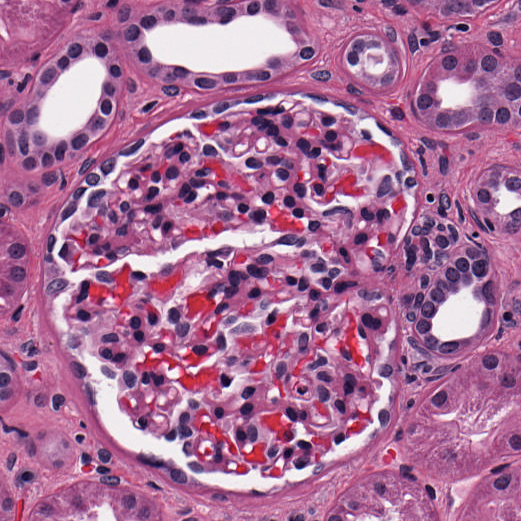

Supplement: Supplementary file 2 — Supplementary material [file mmc1.zip › verification/C3L-00969-27 (1, x=27792, y=10050, w=521, h=521).png]

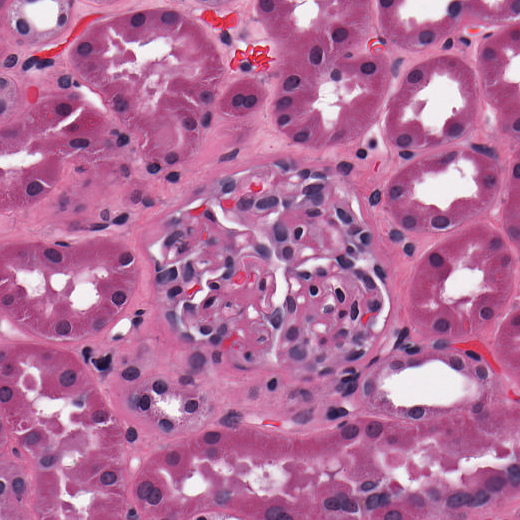

Supplement: Supplementary file 2 — Supplementary material [file mmc1.zip › verification/C3L-00970-27 (1, x=6569, y=5470, w=520, h=520).png]

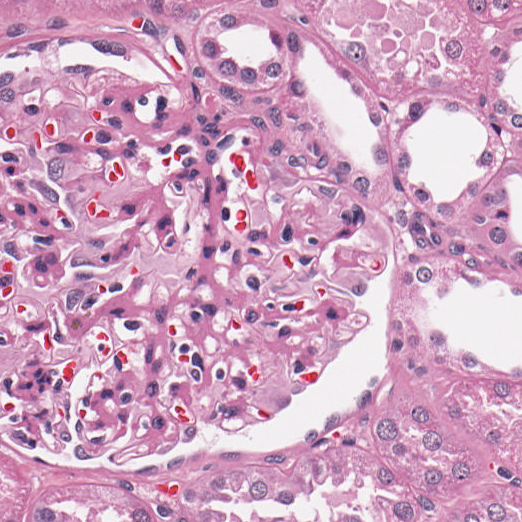

Supplement: Supplementary file 2 — Supplementary material [file mmc1.zip › verification/C3L-00976-28 (1, x=13603, y=11812, w=522, h=522).png]

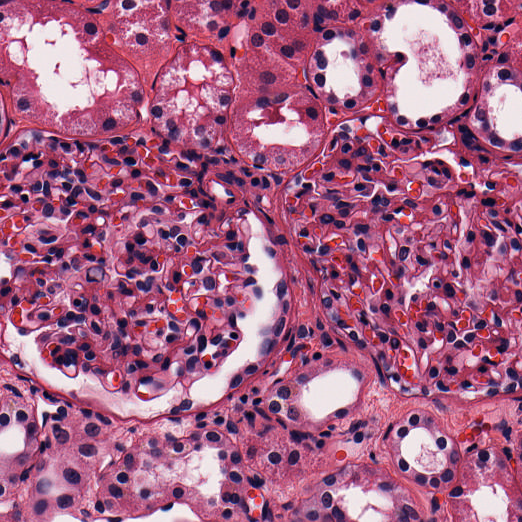

Supplement: Supplementary file 2 — Supplementary material [file mmc1.zip › verification/C3L-00980-26 (1, x=32696, y=15569, w=522, h=522).png]

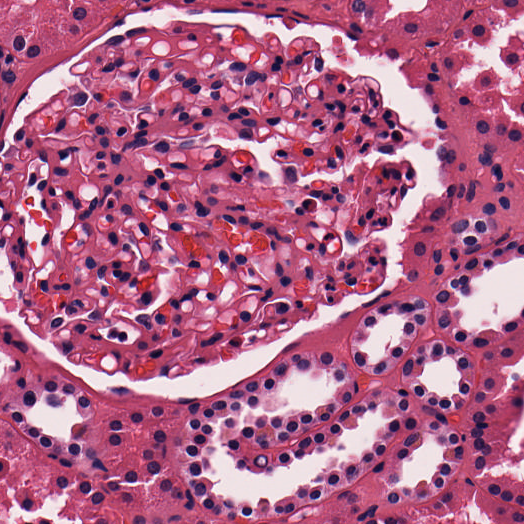

Supplement: Supplementary file 2 — Supplementary material [file mmc1.zip › verification/C3L-00981-26 (1, x=10477, y=17815, w=524, h=524).png]

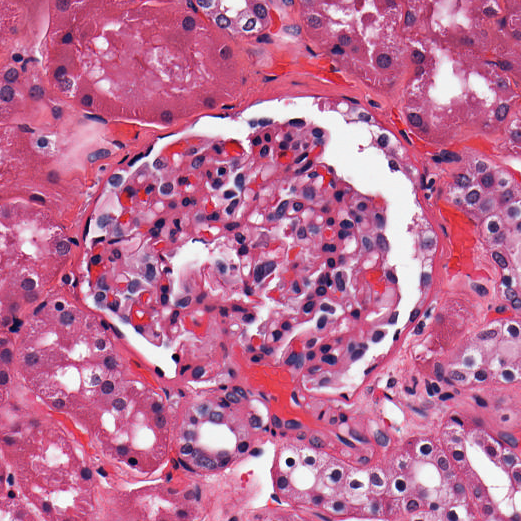

Supplement: Supplementary file 2 — Supplementary material [file mmc1.zip › verification/C3L-00982-26 (1, x=24535, y=19172, w=521, h=521).png]

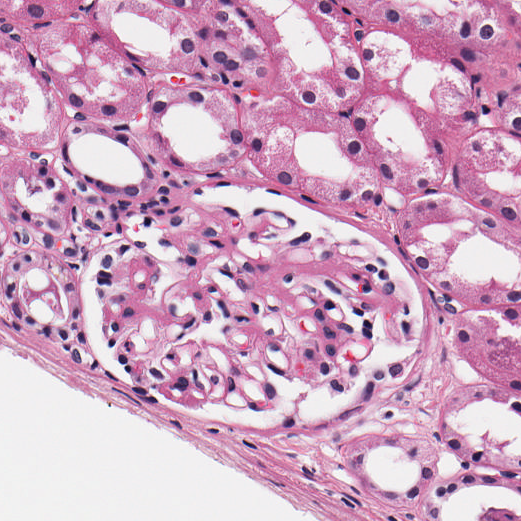

Supplement: Supplementary file 2 — Supplementary material [file mmc1.zip › verification/C3L-01033-25 (1, x=15068, y=25743, w=521, h=521).png]

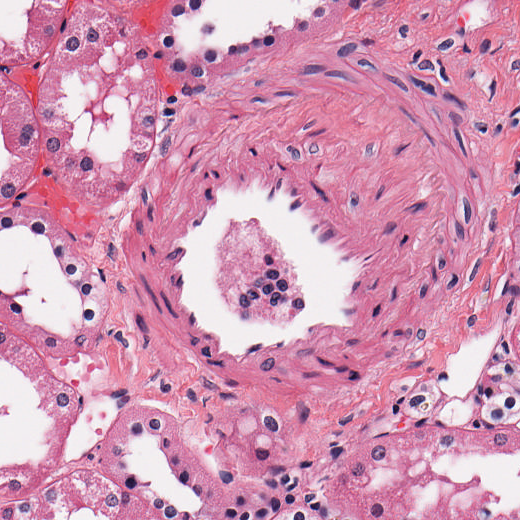

Supplement: Supplementary file 2 — Supplementary material [file mmc1.zip › verification/C3L-01102-26 (1, x=16594, y=16408, w=520, h=520).png]

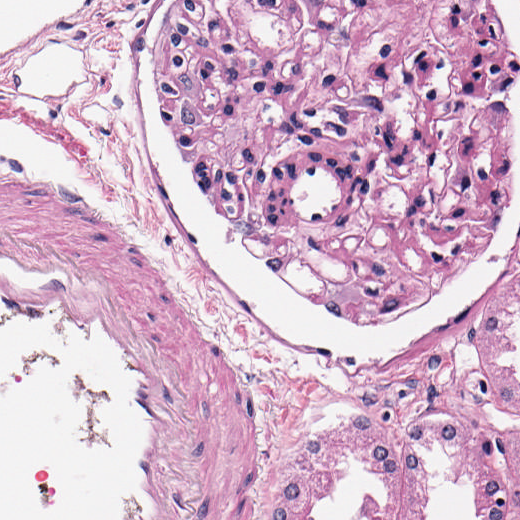

Supplement: Supplementary file 2 — Supplementary material [file mmc1.zip › verification/C3L-01106-26 (1, x=3617, y=22687, w=520, h=520).png]

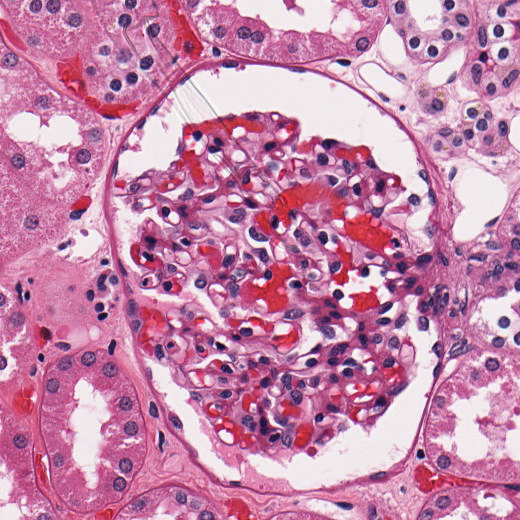

Supplement: Supplementary file 2 — Supplementary material [file mmc1.zip › verification/C3L-01107-27 (1, x=11416, y=7656, w=520, h=520).png]

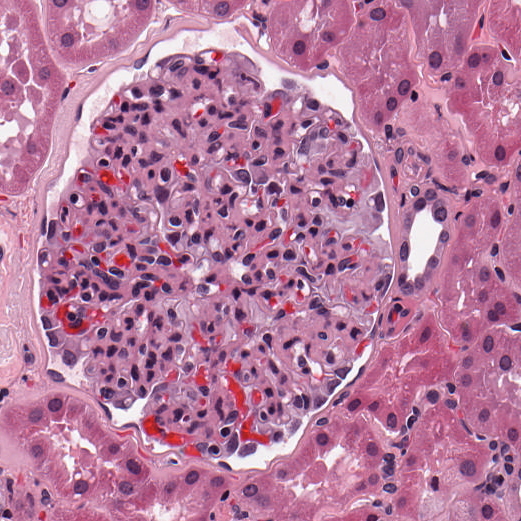

Supplement: Supplementary file 2 — Supplementary material [file mmc1.zip › verification/C3L-01279-26 (1, x=18511, y=11288, w=521, h=521).png]

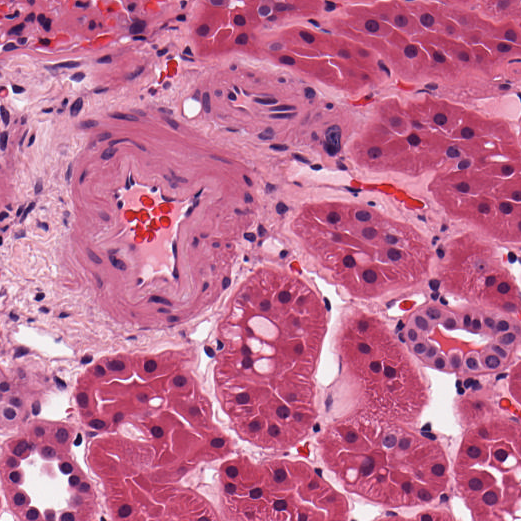

Supplement: Supplementary file 2 — Supplementary material [file mmc1.zip › verification/C3L-01281-26 (1, x=8627, y=2783, w=521, h=521).png]

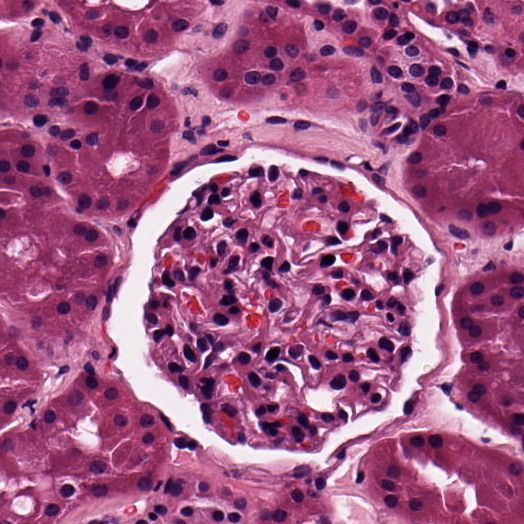

Supplement: Supplementary file 2 — Supplementary material [file mmc1.zip › verification/C3L-01287-26 (1, x=22379, y=19169, w=524, h=524).png]

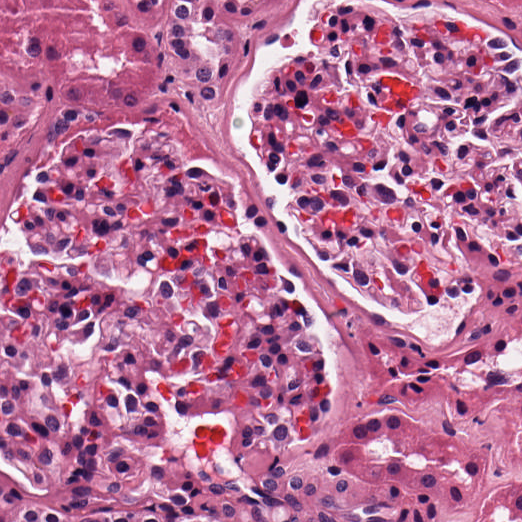

Supplement: Supplementary file 2 — Supplementary material [file mmc1.zip › verification/C3L-01302-26 (1, x=10016, y=11925, w=522, h=522).png]

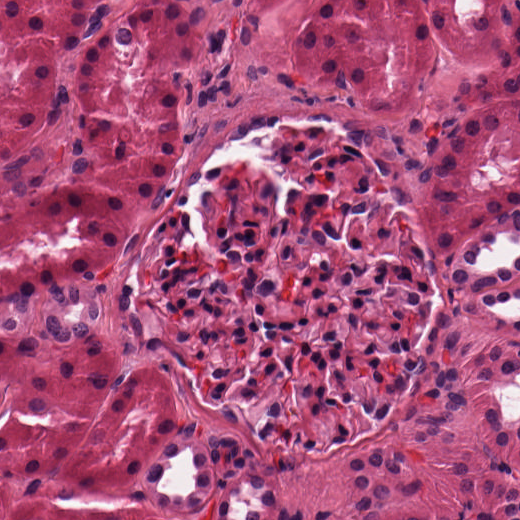

Supplement: Supplementary file 2 — Supplementary material [file mmc1.zip › verification/C3L-01313-26 (1, x=15671, y=5516, w=520, h=520).png]

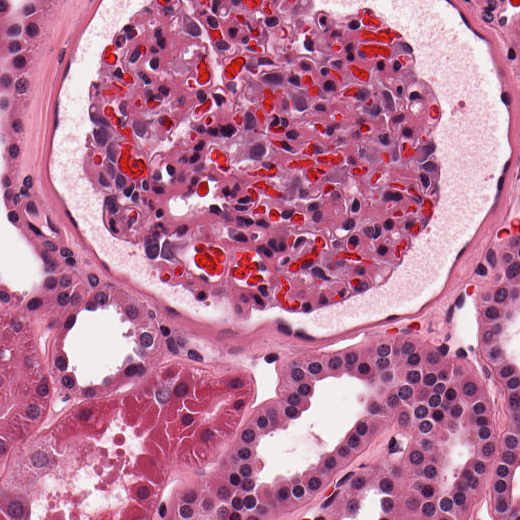

Supplement: Supplementary file 2 — Supplementary material [file mmc1.zip › verification/C3L-01603-26 (1, x=11023, y=8817, w=520, h=520).png]

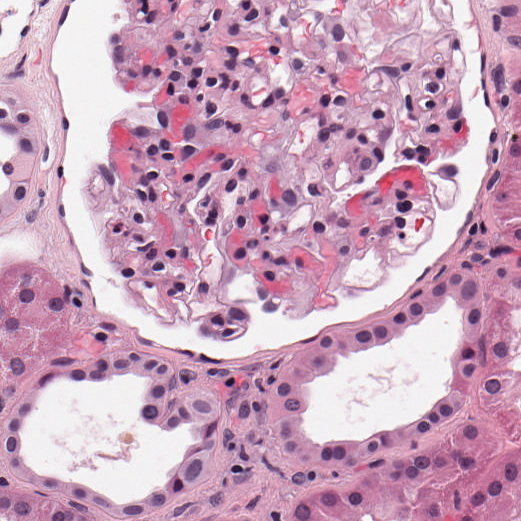

Supplement: Supplementary file 2 — Supplementary material [file mmc1.zip › verification/C3L-01665-26 (1, x=17504, y=3402, w=521, h=521).png]

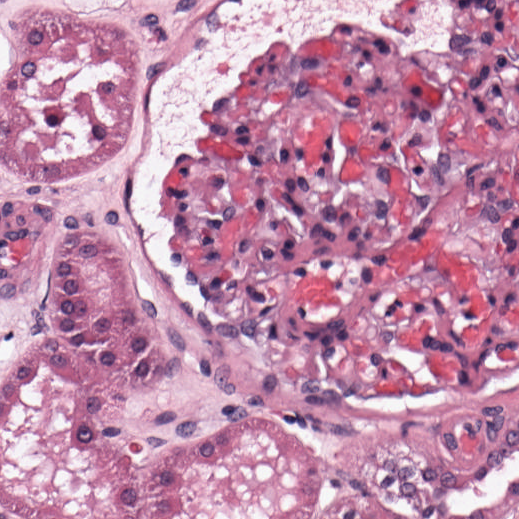

Supplement: Supplementary file 2 — Supplementary material [file mmc1.zip › verification/C3L-01676-27 (1, x=9649, y=13538, w=519, h=519).png]

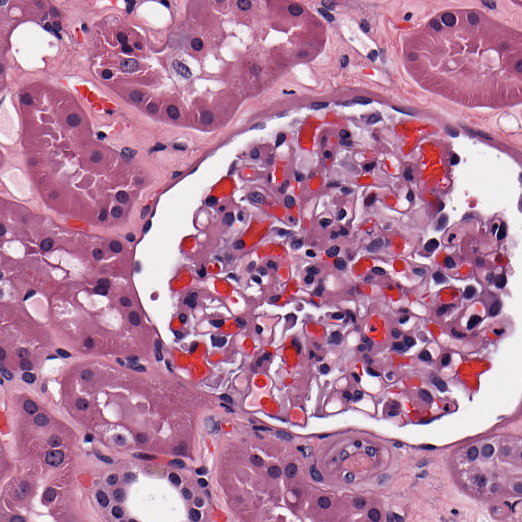

Supplement: Supplementary file 2 — Supplementary material [file mmc1.zip › verification/C3L-01681-29 (1, x=16484, y=32009, w=522, h=522).png]

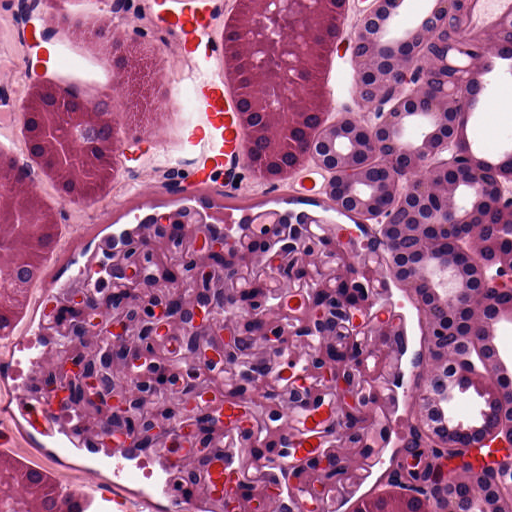

Supplement: Supplementary file 2 — Supplementary material [file mmc1.zip › verification/C3L-01836-26 (1, x=27108, y=27130, w=512, h=512).png]

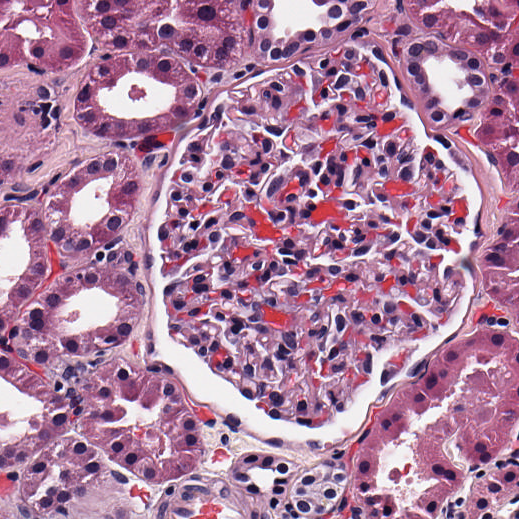

Supplement: Supplementary file 2 — Supplementary material [file mmc1.zip › verification/C3L-01869-26 (1, x=20311, y=12960, w=519, h=519).png]

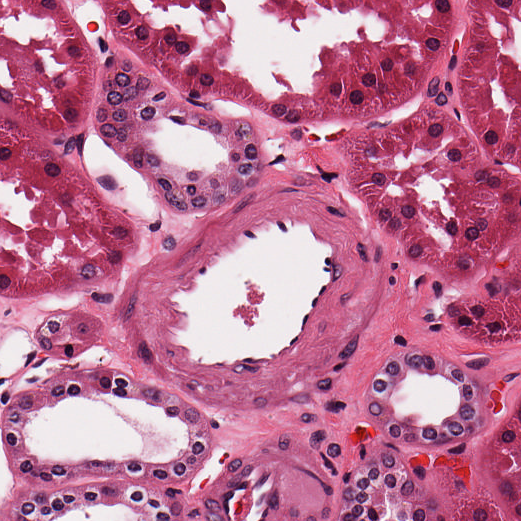

Supplement: Supplementary file 2 — Supplementary material [file mmc1.zip › verification/C3L-01882-26 (1, x=6628, y=16570, w=521, h=521).png]

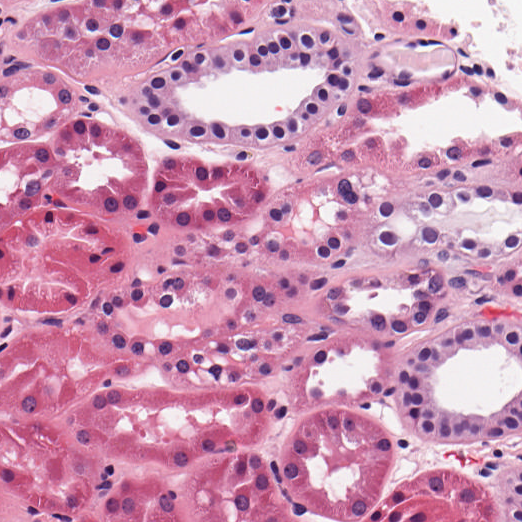

Supplement: Supplementary file 2 — Supplementary material [file mmc1.zip › verification/C3L-02220-26 (1, x=19445, y=18685, w=522, h=522).png]

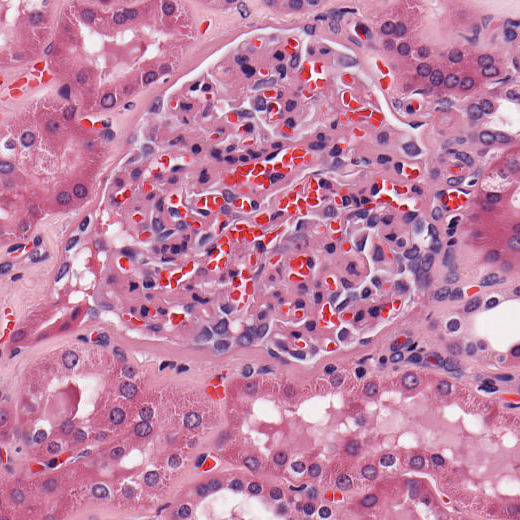

Supplement: Supplementary file 2 — Supplementary material [file mmc1.zip › verification/C3L-02346-26 (1, x=24850, y=25344, w=520, h=520).png]

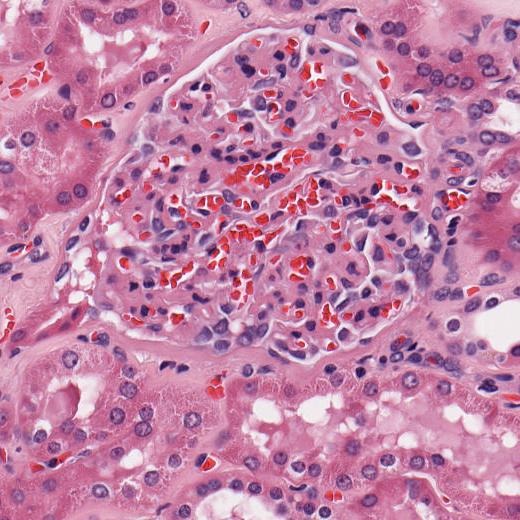

Supplement: Supplementary file 2 — Supplementary material [file mmc1.zip › verification/project/data/100/thumbnail.jpg]

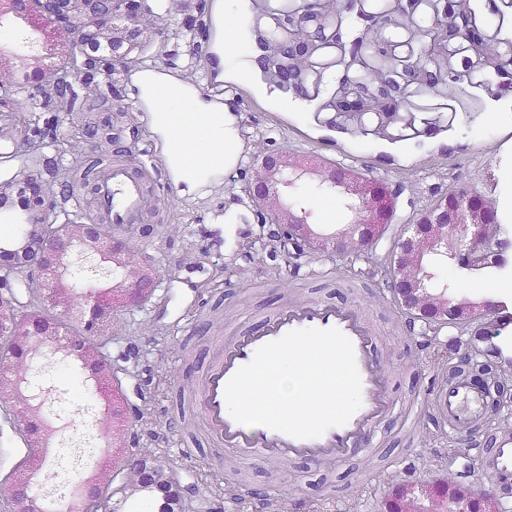

Supplement: Supplementary file 2 — Supplementary material [file mmc1.zip › verification/project/data/48/thumbnail.jpg]

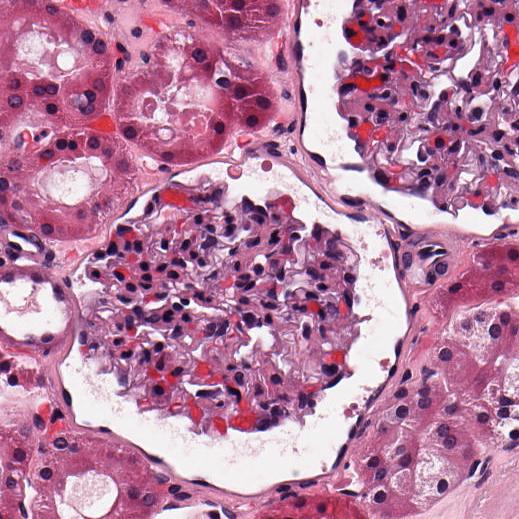

Supplement: Supplementary file 2 — Supplementary material [file mmc1.zip › verification/project/data/51/thumbnail.jpg]

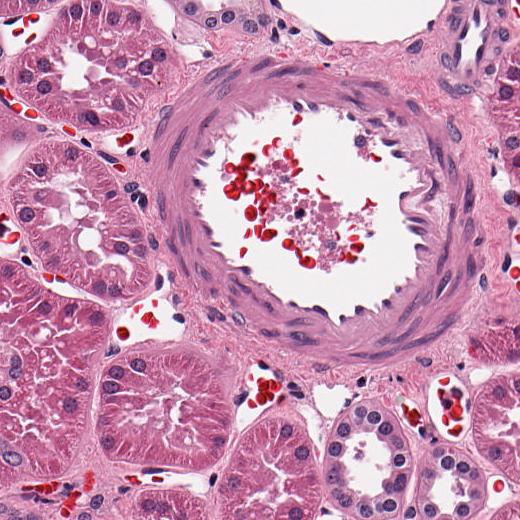

Supplement: Supplementary file 2 — Supplementary material [file mmc1.zip › verification/project/data/52/thumbnail.jpg]

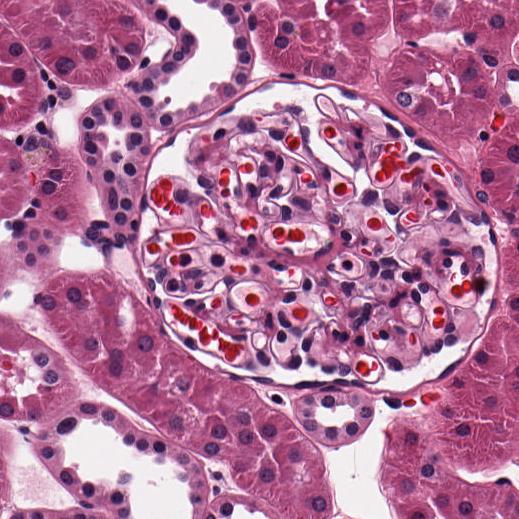

Supplement: Supplementary file 2 — Supplementary material [file mmc1.zip › verification/project/data/53/thumbnail.jpg]

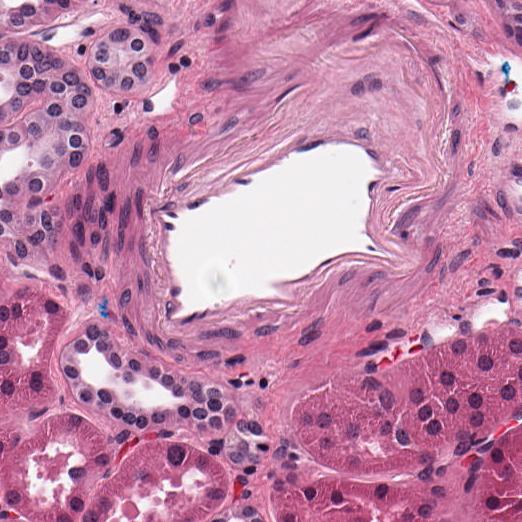

Supplement: Supplementary file 2 — Supplementary material [file mmc1.zip › verification/project/data/54/thumbnail.jpg]

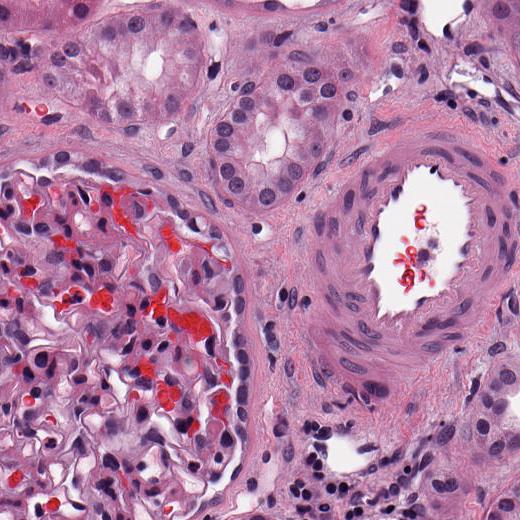

Supplement: Supplementary file 2 — Supplementary material [file mmc1.zip › verification/project/data/55/thumbnail.jpg]

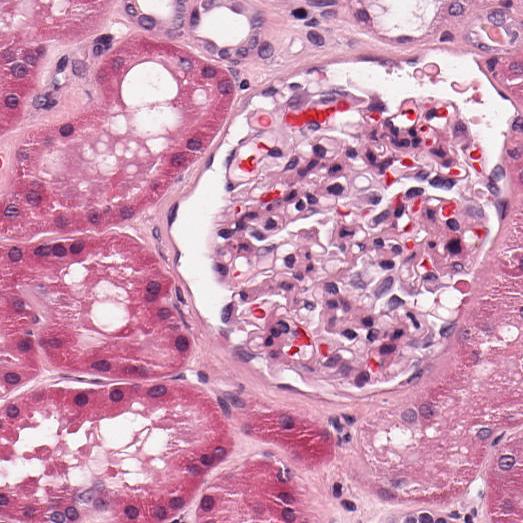

Supplement: Supplementary file 2 — Supplementary material [file mmc1.zip › verification/project/data/56/thumbnail.jpg]

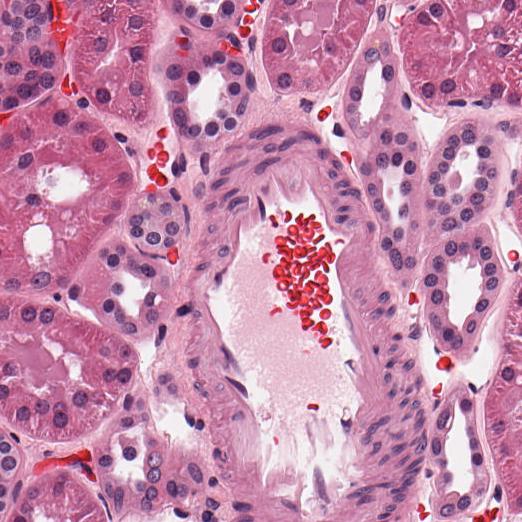

Supplement: Supplementary file 2 — Supplementary material [file mmc1.zip › verification/project/data/57/thumbnail.jpg]

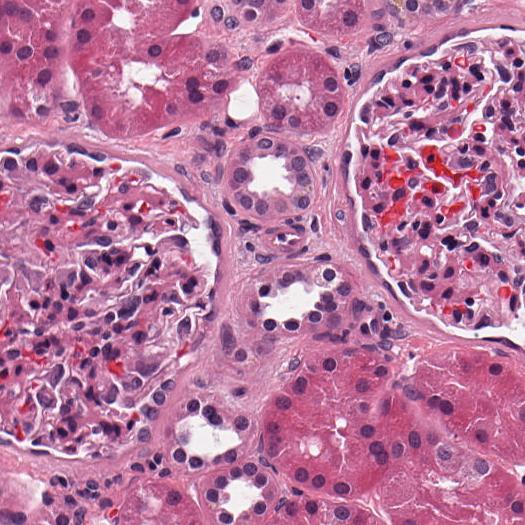

Supplement: Supplementary file 2 — Supplementary material [file mmc1.zip › verification/project/data/58/thumbnail.jpg]

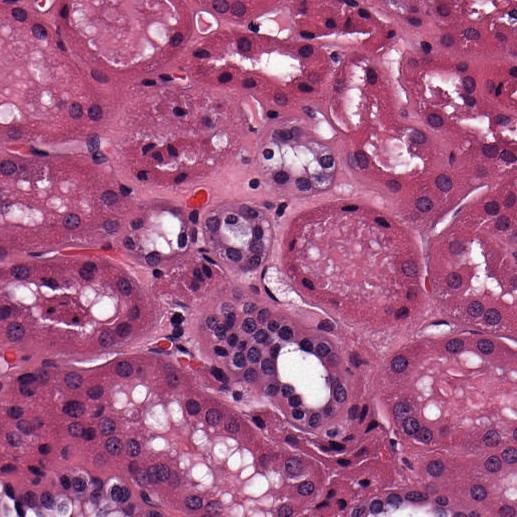

Supplement: Supplementary file 2 — Supplementary material [file mmc1.zip › verification/project/data/59/thumbnail.jpg]

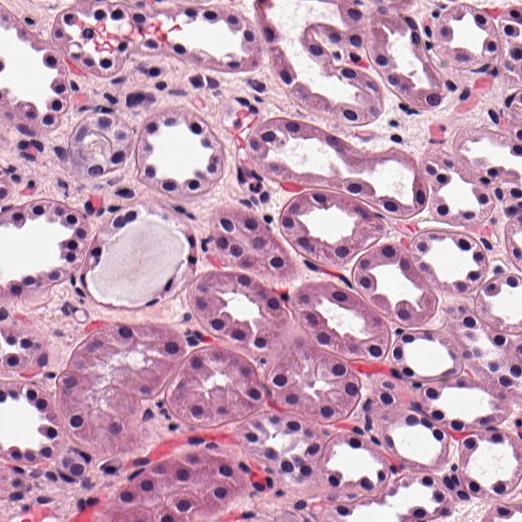

Supplement: Supplementary file 2 — Supplementary material [file mmc1.zip › verification/project/data/60/thumbnail.jpg]

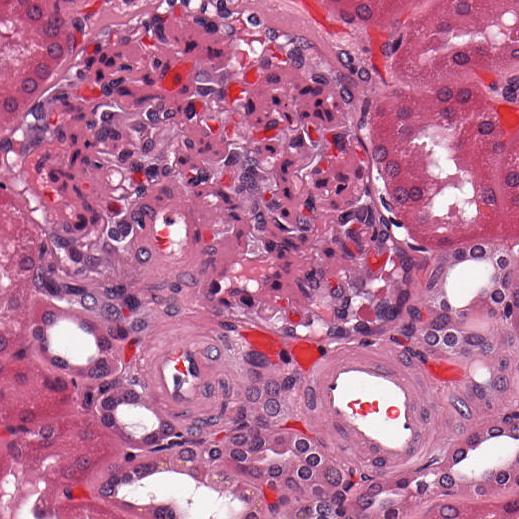

Supplement: Supplementary file 2 — Supplementary material [file mmc1.zip › verification/project/data/61/thumbnail.jpg]

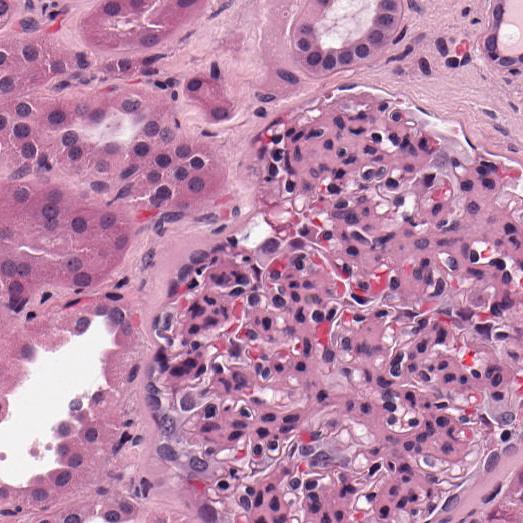

Supplement: Supplementary file 2 — Supplementary material [file mmc1.zip › verification/project/data/62/thumbnail.jpg]

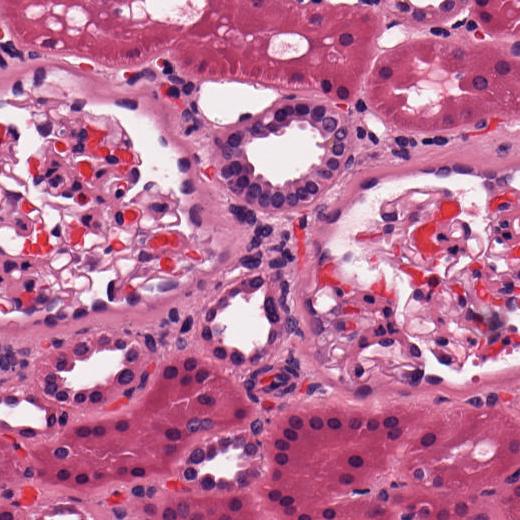

Supplement: Supplementary file 2 — Supplementary material [file mmc1.zip › verification/project/data/63/thumbnail.jpg]

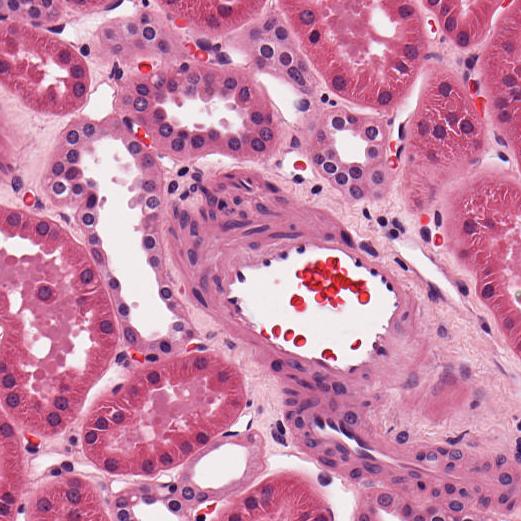

Supplement: Supplementary file 2 — Supplementary material [file mmc1.zip › verification/project/data/64/thumbnail.jpg]

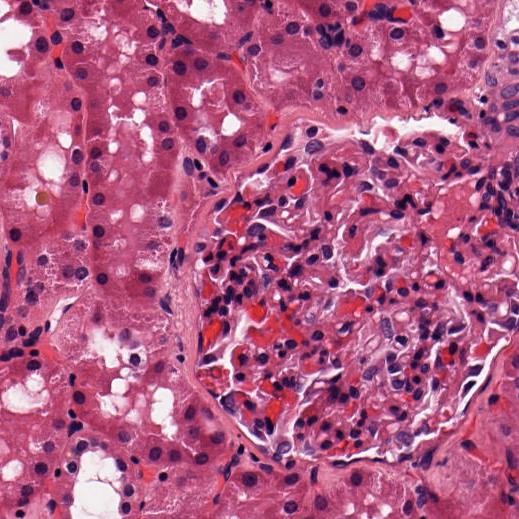

Supplement: Supplementary file 2 — Supplementary material [file mmc1.zip › verification/project/data/65/thumbnail.jpg]

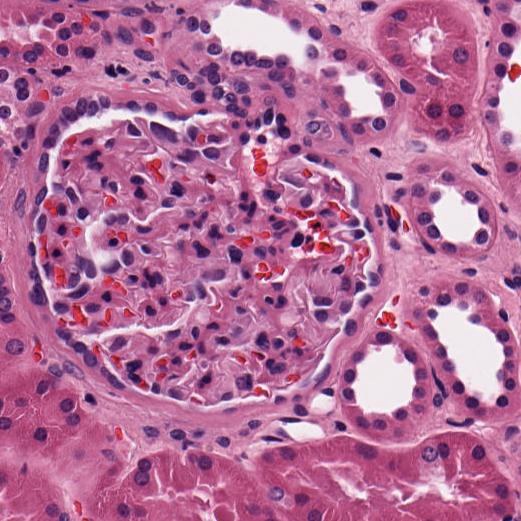

Supplement: Supplementary file 2 — Supplementary material [file mmc1.zip › verification/project/data/66/thumbnail.jpg]

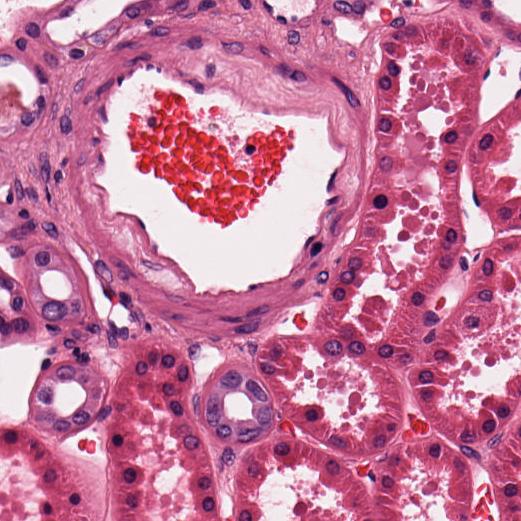

Supplement: Supplementary file 2 — Supplementary material [file mmc1.zip › verification/project/data/67/thumbnail.jpg]

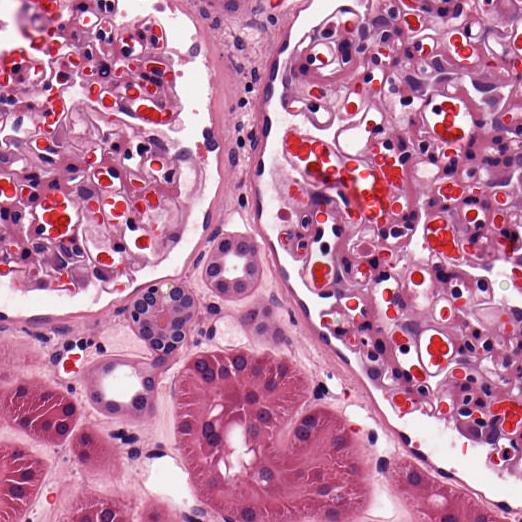

Supplement: Supplementary file 2 — Supplementary material [file mmc1.zip › verification/project/data/68/thumbnail.jpg]

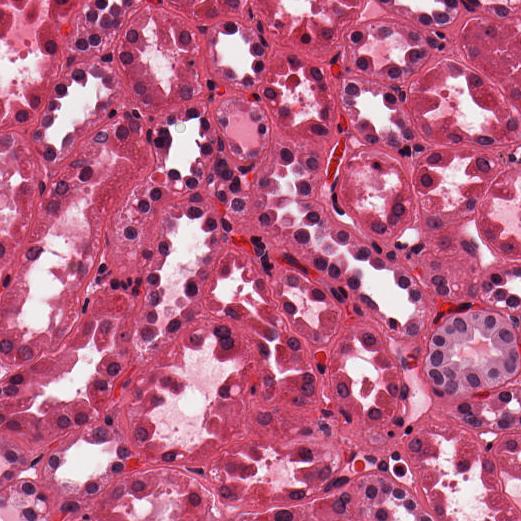

Supplement: Supplementary file 2 — Supplementary material [file mmc1.zip › verification/project/data/69/thumbnail.jpg]

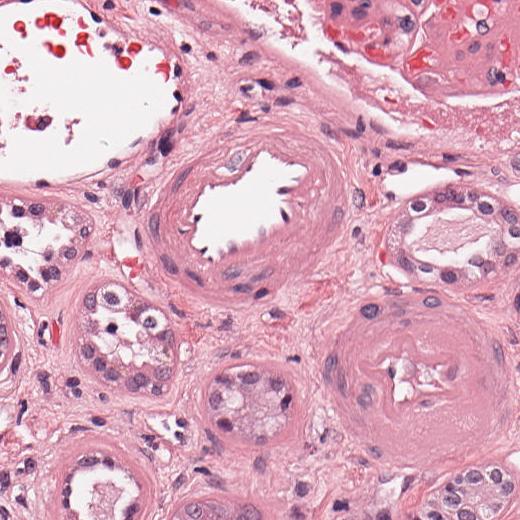

Supplement: Supplementary file 2 — Supplementary material [file mmc1.zip › verification/project/data/70/thumbnail.jpg]

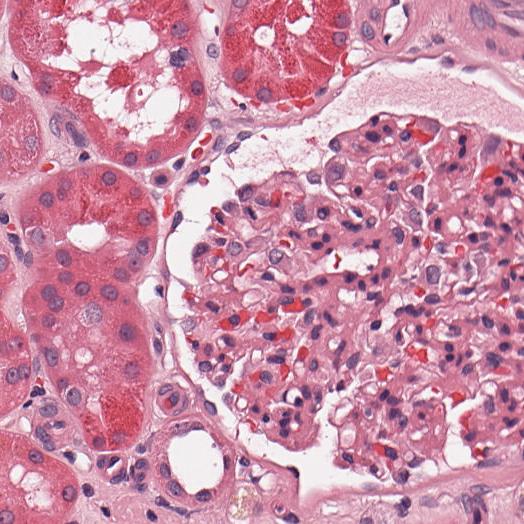

Supplement: Supplementary file 2 — Supplementary material [file mmc1.zip › verification/project/data/71/thumbnail.jpg]

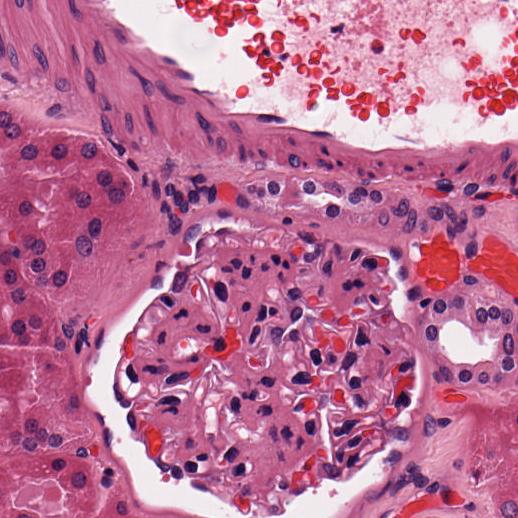

Supplement: Supplementary file 2 — Supplementary material [file mmc1.zip › verification/project/data/72/thumbnail.jpg]

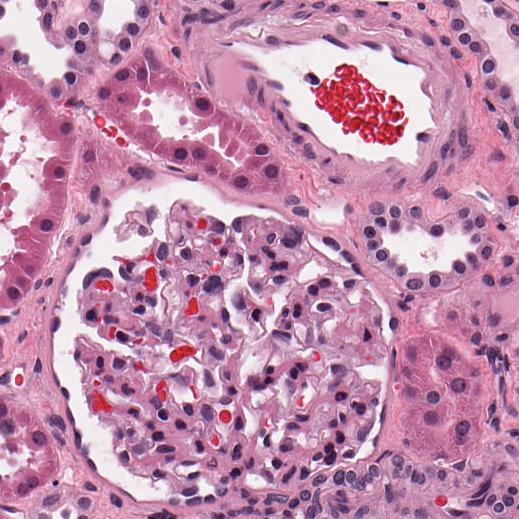

Supplement: Supplementary file 2 — Supplementary material [file mmc1.zip › verification/project/data/73/thumbnail.jpg]

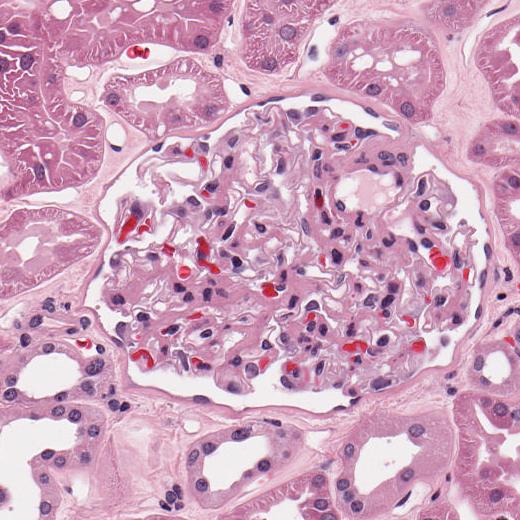

Supplement: Supplementary file 2 — Supplementary material [file mmc1.zip › verification/project/data/74/thumbnail.jpg]

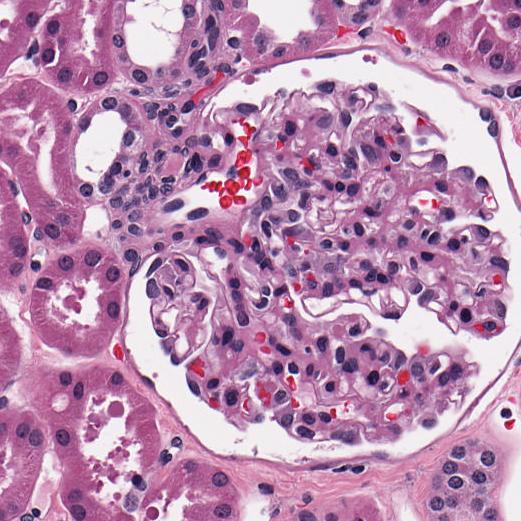

Supplement: Supplementary file 2 — Supplementary material [file mmc1.zip › verification/project/data/75/thumbnail.jpg]

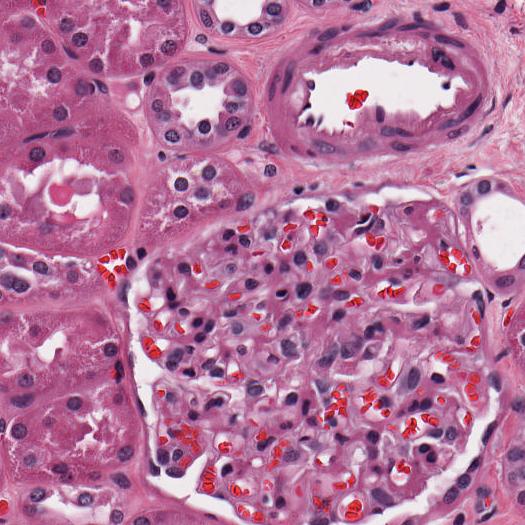

Supplement: Supplementary file 2 — Supplementary material [file mmc1.zip › verification/project/data/76/thumbnail.jpg]

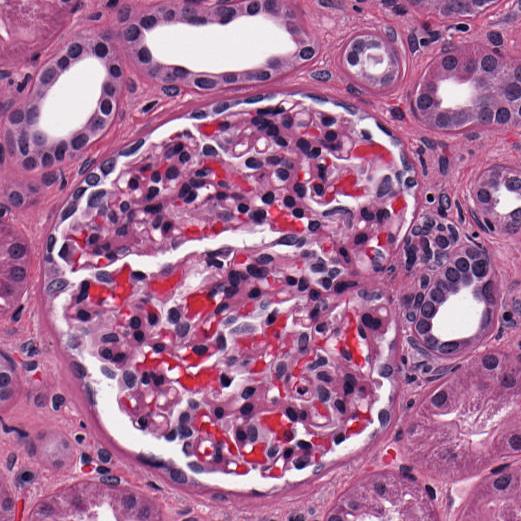

Supplement: Supplementary file 2 — Supplementary material [file mmc1.zip › verification/project/data/77/thumbnail.jpg]

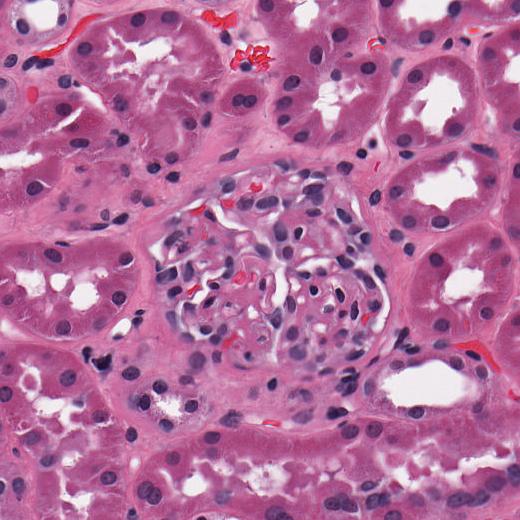

Supplement: Supplementary file 2 — Supplementary material [file mmc1.zip › verification/project/data/78/thumbnail.jpg]

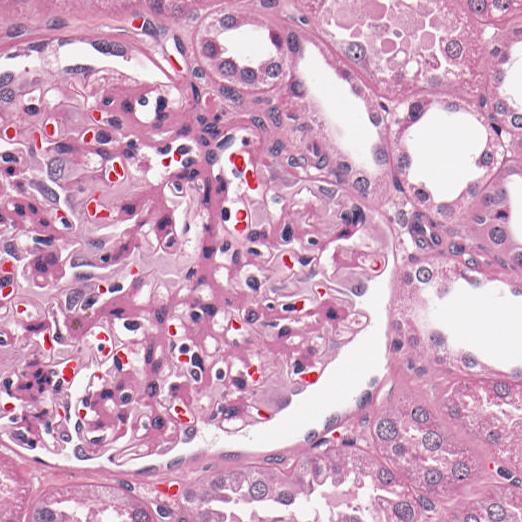

Supplement: Supplementary file 2 — Supplementary material [file mmc1.zip › verification/project/data/79/thumbnail.jpg]

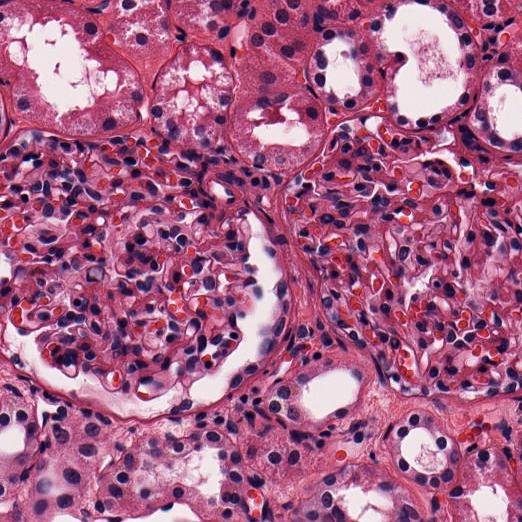

Supplement: Supplementary file 2 — Supplementary material [file mmc1.zip › verification/project/data/80/thumbnail.jpg]

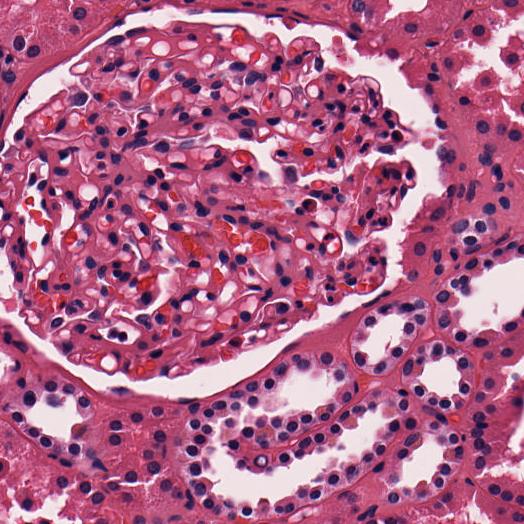

Supplement: Supplementary file 2 — Supplementary material [file mmc1.zip › verification/project/data/81/thumbnail.jpg]

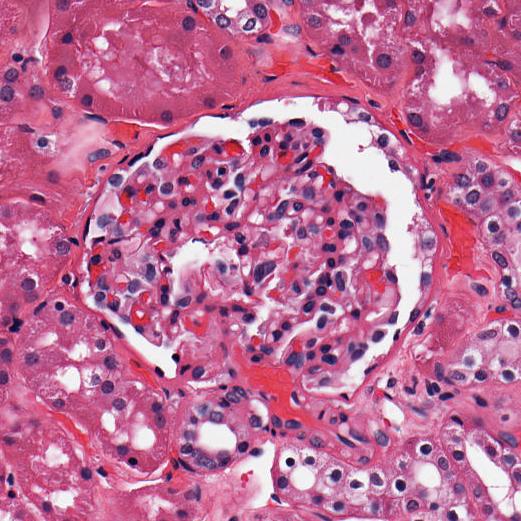

Supplement: Supplementary file 2 — Supplementary material [file mmc1.zip › verification/project/data/82/thumbnail.jpg]

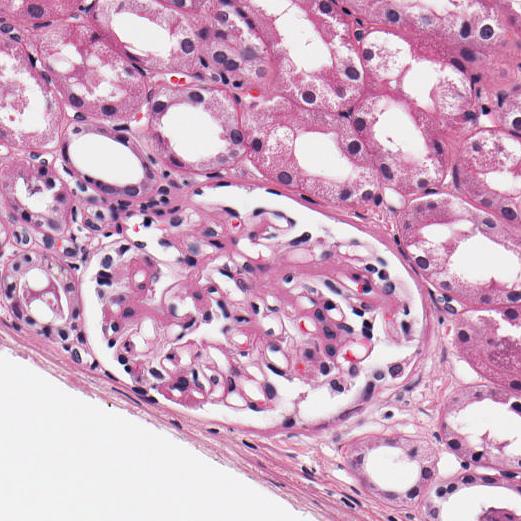

Supplement: Supplementary file 2 — Supplementary material [file mmc1.zip › verification/project/data/83/thumbnail.jpg]

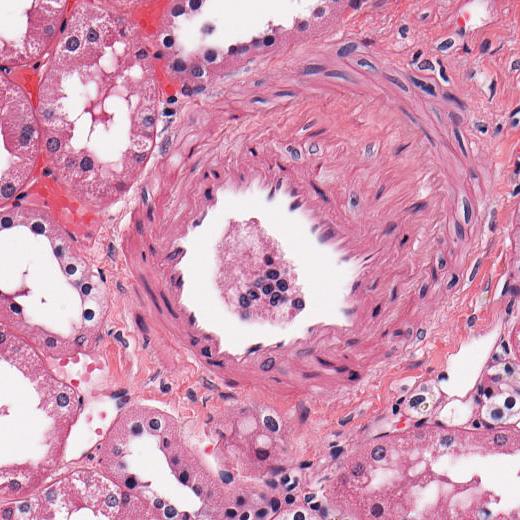

Supplement: Supplementary file 2 — Supplementary material [file mmc1.zip › verification/project/data/84/thumbnail.jpg]

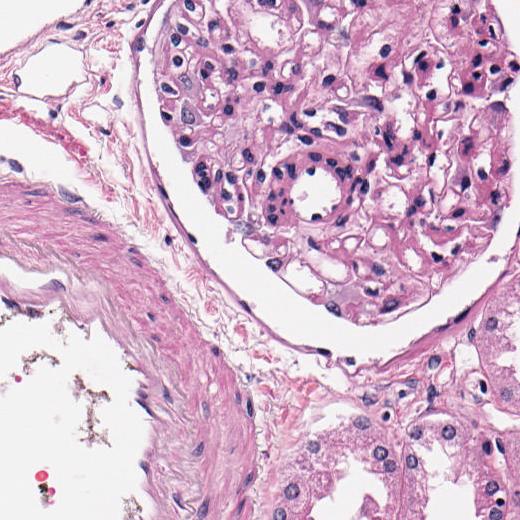

Supplement: Supplementary file 2 — Supplementary material [file mmc1.zip › verification/project/data/85/thumbnail.jpg]
